# Mitigation of deleterious phenotypes in chloroplast-engineered plants accumulating high levels of foreign proteins

**DOI:** 10.1186/s13068-021-01893-2

**Published:** 2021-02-10

**Authors:** Jennifer A. Schmidt, Lubna V. Richter, Lisa A. Condoluci, Beth A. Ahner

**Affiliations:** Department of Biological and Environmental Engineering, Cornell University, Ithaca, NY USA

**Keywords:** Recombinant protein, Cellulase, Tobacco, Chloroplast-engineering, Gibberellic acid, Rubisco, Pleiotropic effects, Commercialization

## Abstract

**Background:**

The global demand for functional proteins is extensive, diverse, and constantly increasing. Medicine, agriculture, and industrial manufacturing all rely on high-quality proteins as major active components or process additives. Historically, these demands have been met by microbial bioreactors that are expensive to operate and maintain, prone to contamination, and relatively inflexible to changing market demands. Well-established crop cultivation techniques coupled with new advancements in genetic engineering may offer a cheaper and more versatile protein production platform. Chloroplast-engineered plants, like tobacco, have the potential to produce large quantities of high-value proteins, but often result in engineered plants with mutant phenotypes. This technology needs to be fine-tuned for commercial applications to maximize target protein yield while maintaining robust plant growth.

**Results:**

Here, we show that a previously developed *Nicotiana tabacum* line, TetC-*cel6*A, can produce an industrial cellulase at levels of up to 28% of total soluble protein (TSP) with a slight dwarf phenotype but no loss in biomass. In seedlings, the dwarf phenotype is recovered by exogenous application of gibberellic acid. We also demonstrate that accumulating foreign protein represents an added burden to the plants’ metabolism that can make them more sensitive to limiting growth conditions such as low nitrogen. The biomass of nitrogen-limited TetC-*cel6*A plants was found to be as much as 40% lower than wildtype (WT) tobacco, although heterologous cellulase production was not greatly reduced compared to well-fertilized TetC-*cel6*A plants. Furthermore, cultivation at elevated carbon dioxide (1600 ppm CO_2_) restored biomass accumulation in TetC-*cel6*A plants to that of WT, while also increasing total heterologous protein yield (mg Cel6A plant^−1^) by 50–70%.

**Conclusions:**

The work reported here demonstrates that well-fertilized tobacco plants have a substantial degree of flexibility in protein metabolism and can accommodate considerable levels of some recombinant proteins without exhibiting deleterious mutant phenotypes. Furthermore, we show that the alterations to protein expression triggered by growth at elevated CO_2_ can help rebalance endogenous protein expression and/or increase foreign protein production in chloroplast-engineered tobacco.

## Background

Proteins are used as enzyme additives in many industrial processes, as antibodies and medical peptides for pharmaceuticals, and as nutritional additives to food and animal feed (reviewed in [1]). These high-value proteins are mainly produced for such purposes using large-scale cultures of bacterial, fungal, or mammalian cells. However, these systems are often expensive to establish, labor-intensive to maintain, prone to contamination, and inflexible to changing market demands [2]. With well-established cultivation practices, a genetically engineered (GE) biomass crop, like tobacco, may represent a low-cost alternative [3–5].

While most GE plants used in commercial applications are generated by manipulation of the nuclear genome, transformation of the chloroplast genome for protein production has several key advantages. For instance, leaf cells contain a high copy number of chloroplast genomes (between 2000 and 20,000 copies per leaf cell [6, 7]), the expression rates of many native chloroplast-encoded genes are high compared to those in the nucleus, and there are no gene-silencing mechanisms in plastids (reviewed in [8]). As a result, chloroplast-engineered plants regularly achieve recombinant protein yields averaging 5–20% of total soluble protein (TSP) with exceptional plastid transformants reaching yields of 40–70% of TSP [3, 9–17].

While these high yields are promising for commercial applications, accumulating a non-native protein to that degree can have unforeseen impacts on the GE plant’s metabolism. Several GE projects have yielded transplastomic plants with undesired mutant phenotypes like delayed growth, infertility, leaf chlorosis, and low biomass yield [10, 15–19]. These impacts are, however, highly variable and often unpredictable prior to establishment of the transplastomic plant. Furthermore, there is rarely subsequent experimentation to investigate the causes of these phenotypes and determine whether they may be ameliorated by factors like fertilization, light levels, or regulatory element selection.

Despite these challenges, some GE plants have been reported to accumulate foreign proteins to as much as 41% of TSP with little or no phenotypic changes. In fact, one group argued that some abundant native proteins, particularly Rubisco, serve as a dynamic nitrogen (N) storage by accumulating to levels greater than is biologically required in wildtype (WT) tobacco; the authors posited that GE plants can reduce the abundance of these native proteins while synthesizing foreign proteins without interrupting native metabolism [9, 20]. In recent work, we showed that the response of plant protein metabolism to foreign protein synthesis is largely dependent on environmental factors. When grown in high-light growth chambers GE tobacco plants accumulated a recombinant cellulase to as much as 38% TSP. While phenotypically indistinguishable from WT plants, these engineered plants redistributed a substantial portion of protein resources away from some native proteins though not Rubisco [3]. However, when these same plants were grown in open fields, they increased total TSP production to effectively maintain constant levels of native proteins while producing exogenous cellulase at 20% of TSP on average [3].

Deleterious mutant phenotypes can develop when foreign protein accumulation depletes native proteins beyond minimum levels required for endogenous metabolism. In these cases, mutant phenotypes may be alleviated by altering environmental conditions to reduce the need for specific endogenous proteins. For example, elevated CO_2_ increases biomass while reducing photorespiration and the demand for photosynthetic machinery, particularly Rubisco, in C3 plants (reviewed in [21, 22]). We, therefore, hypothesize that in situations when synthesis of the recombinant protein strains the host plant’s metabolism, elevated CO_2_ may enable recovery of WT growth.

Mutant phenotypes can also be caused by the inclusion of features in the GE cassette that interfere with native gene expression. Regulatory elements native to the plastid genome of the host species or a closely related species are often used to mediate expression of transgenes because of their compatibility with the host’s gene expression machinery [9, 16, 23–25]. However, the use of these DNA elements can result in potentially deleterious competition for transcriptional or translational factors with endogenous DNA elements within the engineered chloroplasts. For example, the use of the native tobacco *clpP* 5′-untranslated region (UTR) for recombinant NPTII expression caused a pigment-deficient phenotype and a reduction in ClpP1 protease due to competition between the native and transgenic *clpP* 5′UTRs for a specific mRNA maturation factor [26]. Translational regulation by mRNA-binding proteins is common in the chloroplast and not completely understood [27]; thus, constitutive expression of transgenic RNA sequences may have unforeseen consequences on endogenous protein accumulation. In addition, the specific codon usage in the transgenic open reading frames (ORFs) and insertion site choice in the plastid genome can also interfere with native protein expression [28, 29].

Finally, in some cases, the enzymatic activity of the recombinant protein may directly interfere with the host plant’s metabolism. For example, Petersen and Bock suggested that the enzymatic activity of recombinant cell wall-degrading enzymes was responsible for the chlorotic leaves and stunted growth observed in their transformed tobacco [16]. Another group attributed alterations to auxin, gibberellic acid, and cytokinin levels to a chemical interaction of their exogenous β-glucosidase with hormone conjugates [30].

The chloroplast-engineered *Nicotiana tabacum* line used in this study, TetC-*cel6*A, was developed and first characterized by Gray et al. as part of a study examining the effect of the downstream box (DB) on protein expression and, as mentioned above, was also deployed recently in a field study [3]. TetC-*cel6*A expresses an active endoglucanase Cel6A from the soil bacteria *Thermobifida fusca* with a short 5′ DB peptide fusion from the TetC bacterial protein [12]*.* This fused ORF is under control of the T7G10 5′UTR and *psb*A terminator sequences. The cassette was inserted in the *trnI–trnA* intergenic space in the plastid genome inverted repeat region, relying on the strong Prrn promoter for read-through transcription. The highest Cel6A accumulation measured in tissues of the T1 generation TetC-*cel6*A plants was 10.6% of TSP [12] whereas recent work with T2 generation plants averaged 20–40% of TSP [3]. As we explore in this study, plant age, growth conditions, and fertilization regimes can cause variable recombinant protein yields. The resolution of unintended homologous recombination events increases the abundance of accurately transformed plastome copies in subsequent plant generations and, therefore, may contribute to increases in heterologous protein accumulation between early generations [31].

Endoglucanases, along with other cellulases, are used in cellulosic ethanol synthesis from cellulose feedstocks and are also needed for the production of some textiles, paper, detergent, beverages, and animal feeds (reviewed in [32]). Here, we measured Cel6A abundance in T2 TetC-*cel6*A plants (propagated from the original TetC-*cel6*A plants) grown from germination to maturity to explore the underlying causes of transgenic phenotypic deviations from WT tobacco. We also manipulated atmospheric CO_2_ and ammonium nitrate fertilizer application to alter protein resource allocation and study its impact on plant growth and Cel6A yield. Here, we aim to enhance the commercial applicability of GE plants for large-scale production of valuable proteins by using TetC-*cel6*A tobacco as a model to understand and mitigate mutant phenotypes caused by high recombinant protein synthesis.

## Results

### TetC-*cel6*A tobacco exhibited only a minor dwarf phenotype despite substantial accumulation of recombinant cellulase

**Fig. 1:**
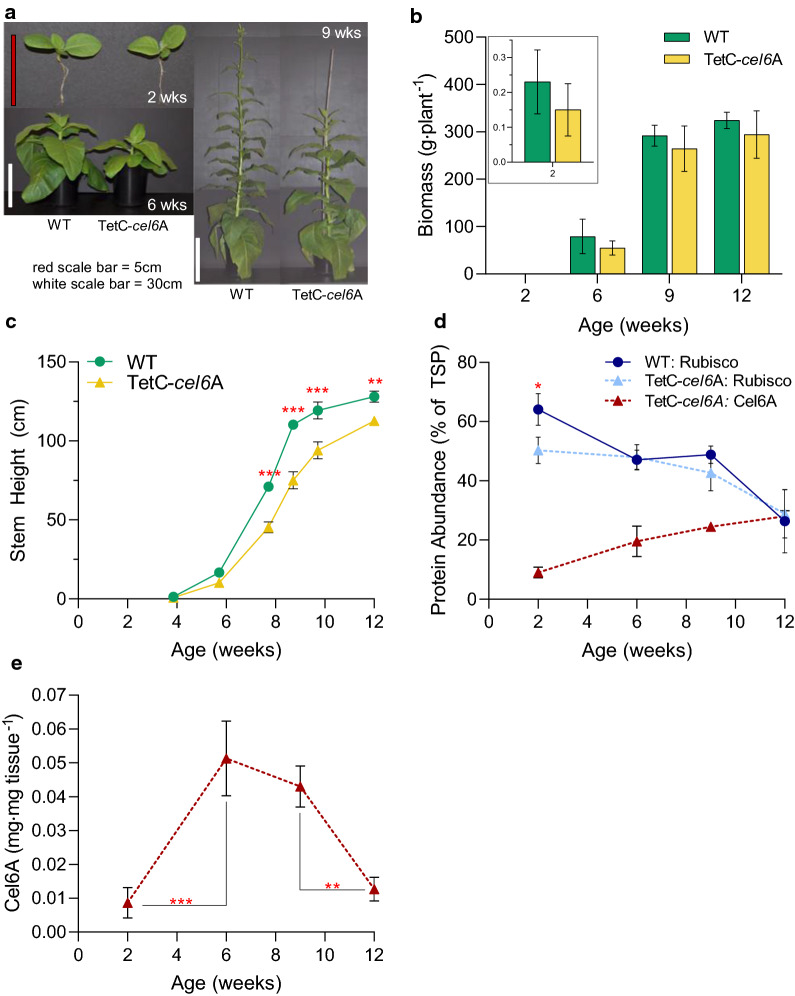
Growth assessment of TetC-*cel6*A tobacco plants from 2 to 12 weeks old. **a** Representative photographs of WT and transgenic plants at 2, 6, and 9 weeks of age. **b** Biomass measurements (*n* = 9 for 2-week harvest, *n* = 4 for 6 and 9-week harvest, and *n* = 3 for 12-week harvest). **c** Measurements of stem elongation from soil to shoot apex (*n* = 4 for all data points except 12 weeks where *n* = 3). **d** Cel6A and Rubisco leaf accumulation as a percent of TSP (*n* = 3). **e** Cel6A yield normalized to mg Cel6A mg^−1^ dry leaf tissue (*n* = 3). Bar heights and data points correspond to the mean and error bars reflect the standard error of the means. The *p*-values for notable comparisons between genotypes and plant age are labeled in red asterisks as follows, *p* < 0.05 (*), *p* < 0.01 (**), and *p* < 0.001 (***). See "Methods" for a description of statistical analyses and Additional file 2: Table S1 contains a full detailed statistics report

The transplastomic and WT tobacco plants were grown in well-fertilized soil pots in growth chambers to document phenotypic differences and to measure recombinant cellulase accumulation as the plant tissue developed over time. TetC-*cel6*A tobacco plants were nearly indistinguishable from WT and produced the same amount of biomass (wet weight) and dry matter at the measured time points (Fig. 1a, b, Additional file 1: Fig. S1a). Leaf area was also not different between the two genotypes (Additional file 1: Fig. S1b). The only altered phenotype in the transgenic plants under these growth conditions was a reduction in stem height of as much as 40% in TetC-*cel6*A plants compared to WT plants after 8 weeks of growth (Fig. 1c).

Cel6A yield as a percent of TSP increased over time in composite tissue samples from 9% Cel6A of TSP in 2-week-old seedlings to 28% in green leaves of 12-week-old plants (Fig. 1d). In contrast to Cel6A, Rubisco content decreased over time from approximately 50% of TSP in seedlings to 30% in 12-week-old plant tissue. No significant differences in Rubisco between genotypes were noted except for a reduction in Rubisco in 2-week-old TetC-*cel6*A seedlings (Fig. 1d). The transgenic plants maintained similar total protein per plant as WT plants and exhibited the same pattern of reduced total protein due to senescence at 12 weeks (Additional file 3: Fig. S2a).

Cel6A concentration (normalized to tissue biomass) varied as a function of plant age, increasing by a factor of five from 2 to 6 weeks, holding steady for at least 3 weeks (6 to 9 weeks) and then decreasing at 12 weeks (Fig. 1e); however, 9-week-old TetC-*cel6*A plants yielded the most Cel6A (mg plant^−1^) as a combined result of plant size and age-dependent protein levels (Additional file 3: Fig. S2b). Consistent with previous work [3], chamber-grown TetC-*cel6*A plants reduced “other” proteins relative to WT, although this reduction was only statistically significant in 9-week-old plants because of replicate variability in protein measurements at other time points (Additional file 3: Fig. S2c, d).

### Exogenous gibberellic acid application alleviates the delayed germination and dwarf phenotype in TetC-*cel6*A tobacco

**Fig. 2:**
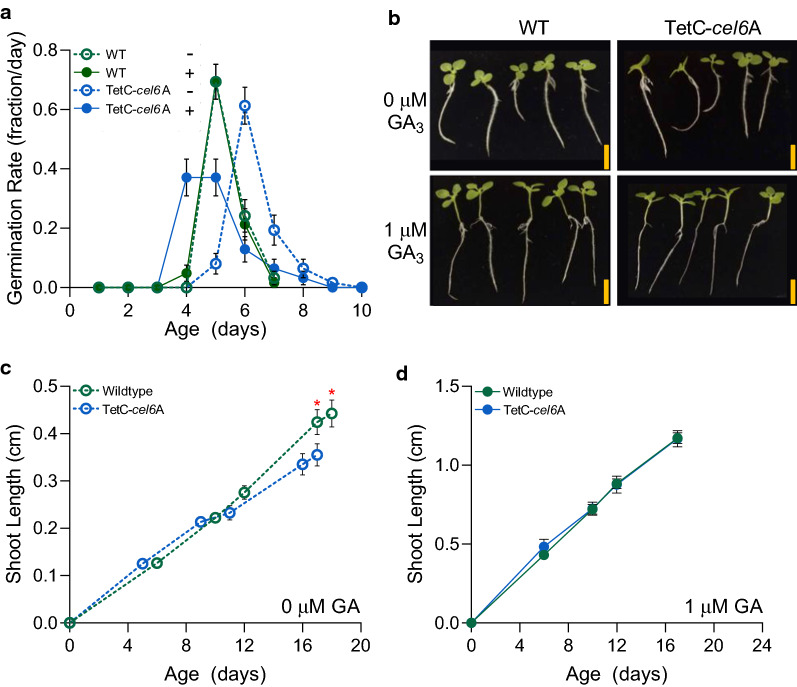
Effect of gibberellic acid on germination and seedling shoot elongation. **a** Germination rate represented as the fraction of the total number of seeds that germinated per day (*n* = 62 independent plants per genotype and treatment). **b** Representative photographs of seedlings approximately 2 weeks post-germination. **c** Shoot elongation of untreated seedlings (*n* = 16 independent biological replicates). **d** Shoot elongation of seedlings treated with 1 µM GA (*n* = 16 independent biological replicates). Data points represent the mean with error bars corresponding to the standard error of the means. The *p*-values for notable comparisons are labeled in red asterisks (*) indicating *p*-values < 0.05. For simplicity not all significant comparisons are depicted on the graphs. See "Methods" for a description of statistical analyses and Additional file 2: Table S1 contains a full detailed statistics report

In addition to the dwarf stem phenotype noted above, we also previously observed a small delay in germination of the TetC-*cel6*A seeds. Combined, those two phenotypes led us to hypothesize that the presence of Cel6A in the GE plant tissue altered gibberellic acid (GA) metabolism because of GA’s role in germination timing and internode elongation in higher plants [33, 34]. Without exogenous GA application, TetC-*cel6*A seeds took on average 6.3 (S.E. ± 0.1) days to germinate on agar plates, a full day longer than WT seeds (Additional file 4: Fig. S3) but with the same profile in the germination rate (Fig. 2a). The addition of 1 µM GA to the plates eliminated the delay on average (5.5 days ± 0.14, Additional file 4: Fig. S3), but the pattern of germination was not the same; 40% of the TetC-*cel6*A seeds germinated a day earlier than most WT seeds, but a subset of the transgenic seeds remained delayed (Fig. 2a). WT seeds with and without the 1 µM GA treatment germinated in the same pattern (Fig. 2a) and with the same average time to germination (5.2 days [S.E. ± 0.07] and 5.3 days [S.E. ± 0.07] after seeding, respectively, Additional file 4: Fig. S3).

The stem length of GA-treated and untreated seedlings was measured over time after germination. Untreated TetC-*cel6*A seedlings began to differentiate from WT at 11 days post-germination, with 15% shorter stems (Fig. 2b, c). This difference in stem elongation increased with time, resulting in transgenic plants that were 20% shorter than WT at 17 days post-germination (Tukey HSD *p*-value < 0.04; Fig. 2b, c). In contrast, when provided with 1 µM GA, the stem length of both TetC-*cel6*A and WT seedlings was indistinguishable (Fig. 2b, d) and more than three times longer than untreated shoots at the end of the experiment (Fig. 2c, d). Thus, in addition to improving germination time, exogenous application of GA also abolished the dwarf stem phenotype observed in untreated TetC-*cel6*A plants.

### Elevated CO_2_ improves growth of TetC-*cel6*A plants when grown in nutrient-limiting conditions

**Fig. 3:**
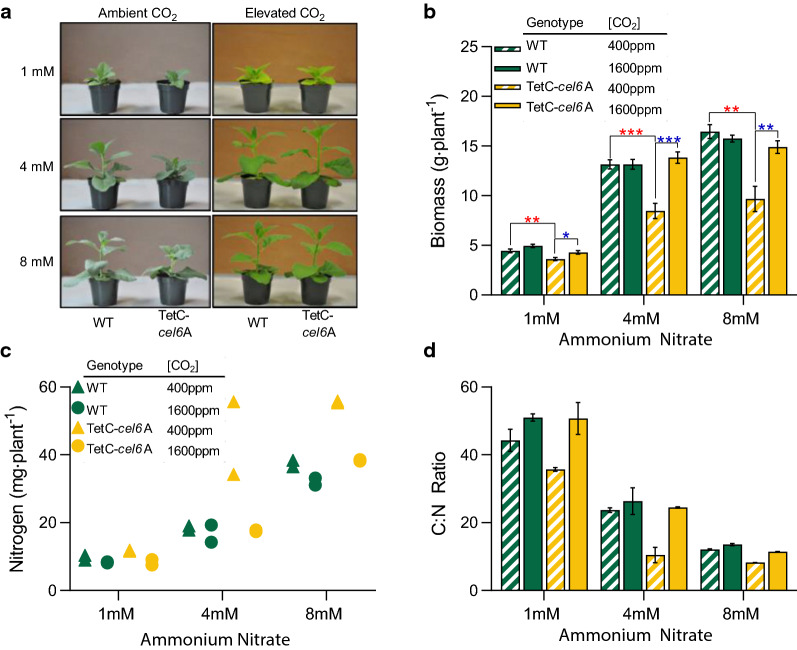
Effect of ammonium nitrate and carbon dioxide on growth of transgenic tobacco. **a** Representative photos of 6-week-old plants. **b** Biomass accumulation (*n* = 6 independent biological replicates). **c** Total leaf nitrogen content (*n* = 2 independent biological replicates). **d** Leaf carbon to nitrogen ratio (*n* = 2 independent biological replicates). Bar heights and data points correspond to the mean and error bars reflect the standard error of the means. The *p*-values for notable comparisons between genotypes (red) and between CO_2_ treatments (blue) were labeled with as follows, *p* < 0.05 (*), *p* < 0.01 (**), and *p* < 0.001 (***). See "Methods" for a description of statistical analyses and Additional file 2: Table S1 contains a full detailed statistics report

We hypothesized that the burden of foreign protein synthesis may have a greater impact on plant phenotype under nutrient-limiting conditions. TetC-*cel6*A and WT tobacco were grown on an inert vermiculite media and fertilized with 1 mM, 4 mM, and 8 mM ammonium nitrate under ambient and elevated CO_2_. At ambient CO_2_, WT biomass increased significantly with each increase in ammonium nitrate as expected, whereas the biomass of TetC-*cel6*A tobacco increased from 1 to 4 mM but no further increase was observed at 8 mM (Fig. 3a, b). Furthermore, TetC-*cel6*A tobacco plants were smaller relative to WT at all ammonium nitrate concentrations (Fig. 3a, b) but, in contrast to our expectations, biomass reductions were correlated with greater N availability: transgenic plants receiving the most ammonium nitrate were the most reduced in biomass relative to WT (41% less at 8 mM; Fig. 3a, b). This result suggests that there may be other stresses or limitations imposed by growth on the vermiculite medium contributing to the low biomass phenotype of TetC-*cel6*A.

The stunted phenotype observed in the transgenic plants was eliminated by elevating atmospheric CO_2_ concentration to 1600 ppm. At this higher level, the fresh weight per plant of the transgenic plants increased to match WT biomass levels at every ammonium nitrate concentration (Fig. 3a, b). However, the dry weight of the TetC-*cel6*A tobacco still lagged those of WT plants (Additional file 5: Fig. S4 a & b), suggesting that some portion of the fresh weight biomass recovery caused by elevated CO_2_ was due to an increase in tissue water content (Additional file 5: Fig. S4 a, b). The biomass and dry weight of WT tobacco exhibited very little response to elevated CO_2_; a statistically significant increase was only noted in WT plants fertilized with 4 mM ammonium nitrate (Additional file 5: Fig. S4a).

While increasing ammonium nitrate treatment increased total N accumulated per plant in both plant genotypes, at ambient CO_2_, transgenic plants accumulated more N than WT at both higher N treatments (Fig. 3c). This difference was abolished at the higher CO_2_ level (Fig. 3c). Consistent with this finding, C:N ratios were significantly lower in TetC*-cel6*A plants compared to WT at ambient CO_2_ concentration, likely reflecting a combination of reduced carbon fixation and the comparatively higher levels of N assimilation (Fig. 3d). At elevated CO_2_, the C:N ratio in TetC*-cel6*A plants are identical to those of WT plants (Fig. 3d), which we hypothesize is due to a recovery of native plant carbon metabolism in the transgenic plants.

### Elevated CO_2_ enables reallocation of protein resources, increasing recombinant cellulase production and bolstering accumulation of native proteins

**Fig. 4:**
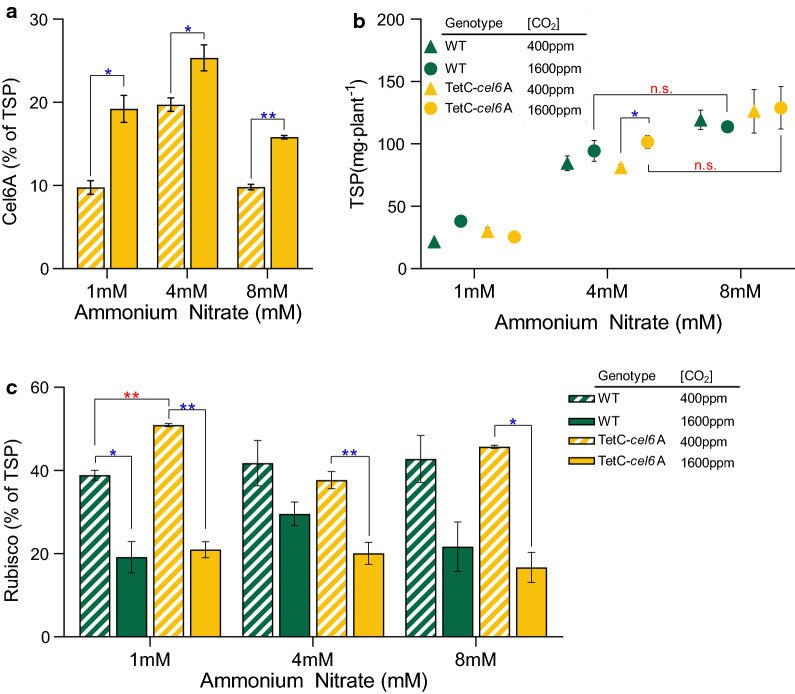
Alteration in recombinant cellulase and Rubisco abundance with varied ammonium nitrate and carbon dioxide levels. **a** TSP accumulation. **b** Cel6A accumulation measured as a percent of TSP. **c** Rubisco accumulation measured as a percent of TSP. Bar heights and data points correspond to the mean and error bars reflect the standard error of the means (*n* = 3 independent biological replicates). The *p*-values for notable comparisons between genotypes (red) and between CO_2_ treatment (blue) were labeled as follows, *p* < 0.05 (*) and *p* < 0.01 (**). Comparisons that were not significantly different were denoted with “n.s.” in red. See "Methods" for a description of statistical analyses and Additional file 2: Table S1 contains a full detailed statistics report

Both N availability and CO_2_ treatment had a significant impact on TetC-*cel6*A accumulation. In ambient CO_2_, the TetC-*cel6*A tobacco treated with both 1 mM and 8 mM ammonium nitrate yielded 10% Cel6A of TSP, while plants treated with 4 mM ammonium nitrate accumulated Cel6A to 20% of TSP (Fig. 4a). Increased CO_2_ led to higher Cel6A yields at all ammonium nitrate levels. The highest concentration was once again measured in plants treated with 4 mM ammonium nitrate, averaging 25% Cel6A of TSP (Fig. 4a), which exceeded the accumulation measured in similarly aged well-fertilized soil-grown plants at ambient CO_2_ (Fig. 1d). Furthermore, total leaf TSP (per plant) was similar between genotypes and generally unresponsive to CO_2_ treatment (Fig. 4b), except at 4 mM ammonium nitrate where elevated CO_2_ led to 25% percent more whole plant protein. The slightly higher TSP in the 8 mM ammonium nitrate TetC-*cel6*A plants resulted in similar absolute levels of Cel6A (mg plant^−1^) as those treated with 4 mM ammonium nitrate (Additional file 5: Fig. S4 c).

We also measured Rubisco abundance (% TSP) in all ammonium nitrate treatments with ambient and elevated CO_2_. When grown in ambient CO_2_, transformed tobacco and WT tissues maintained similar Rubisco levels at 40–50% TSP (Fig. 4c). At elevated CO_2_, plants of both genotypes reduced Rubisco abundance to 19–30% of TSP in WT and 17–20% in TetC-*cel6*A (Fig. 4c).

**Fig. 5:**
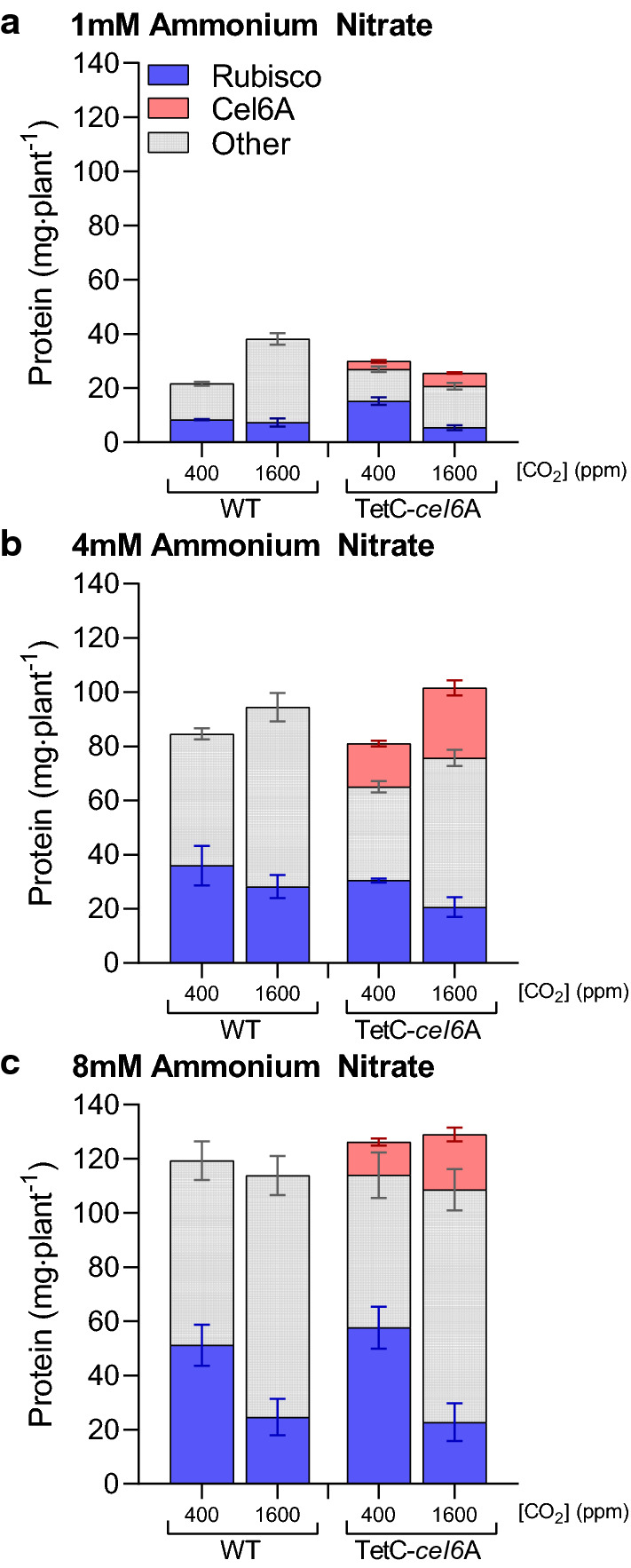
Variation in protein resource allocation dependent on ammonium nitrate and carbon dioxide content. Leaf TSP divided into Rubisco, Cel6A, and all “Other” endogenous soluble proteins for 1 mM (**a**), 4 mM (**b**), and 8 mM (**c**) ammonium nitrate. See "Methods" for a description of statistical analyses and Additional file 2: Table S1 contains a full detailed statistics report

Production of Cel6A without a corresponding increase in whole plant TSP indicates that some subset of endogenous leaf soluble proteins must be down-regulated to accommodate heterologous cellulase synthesis in TetC-*cel6*A tobacco (Fig. 5, “Other”). Overall, at ambient CO_2_, TetC-*cel6*A tobacco averaged 25% less “Other” proteins than WT tobacco. Meanwhile, at elevated CO_2_, it is clear that reduced Rubisco levels lead to higher Cel6A accumulation, but transformants also increased their pool of “Other” proteins, suggesting that a recovery of necessary native protein expression in the TetC-*cel6*A plants enabled the return to WT growth.

## Discussion

Successful use of plastid engineering for commercial production of proteins requires the generation of plants that have both high recombinant protein accumulation as well as reliable growth. However, chloroplast transformation sometimes unpredictably results in plants with mutant phenotypes like chlorotic leaves, stunted growth, and reduced fertility, even when grown in the well-managed conditions of growth chambers and greenhouses [10, 15–19]. These mutant phenotypes can be caused by either broadly overwhelming the host plant’s protein metabolism or by more specific perturbations driven by particular recombinant proteins and/or transgenic regulatory elements.

We and others have shown that chloroplast-engineered tobacco has a sizable capacity to reallocate resources from endogenous proteins with no impact on plant growth; however, at some point, reduced levels of native proteins will compromise physiological processes. For example, Oey and colleagues argued that recombinant PlyGBS production to 70% of TSP exhausted native protein metabolism and severely stunted plant growth [15]. Recent work with the same TetC-*cel6*A tobacco transformant used here, along with work from several other groups, have demonstrated that plastid-engineered plants can accumulate recombinant proteins up to 40% of TSP without deleterious phenotypes [3, 9, 10]. This buffering capacity to accommodate foreign protein synthesis is highly dependent on growth conditions, as demonstrated by the reduced biomass relative to WT and lower Cel6A yields with lower N input for the TetC-*cel6*A tobacco.

Additionally, in this nitrogen limitation trial, we had hypothesized that Cel6A accumulation would be directly correlated with ammonium nitrate input. However, while total protein increased with increasing N, plants fertilized with 4 mM ammonium nitrate accumulated twice as much Cel6A, averaging 20% of TSP, as the plants in the 1 mM and 8 mM treatments. In contrast, one previous study found that N fertilization that varied from 0 to 20 mM ammonium nitrate did not impact recombinant protein accumulation as a percent of TSP [9].

We propose that heterologous protein accumulation in response to changes in resource availability may be partially dependent on the foreign protein’s resistance to degradation by the plastid’s native protein degradation machinery. Degradation of heterologous proteins appears to be highly variable and dependent on the properties of the foreign protein of interest [9, 19, 35, 36]. Nitrogen limitation leads to increased protein turnover and/or altered expression of specific proteases in plants to make amino acids available for new protein synthesis [37–39]. Cel6A’s continued presence in 12-week-old plant tissue after the onset of senescence suggests that it may be resistant to degradation and therefore more stable than native plant proteins. It is therefore possible that the high Cel6A yield noted at the intermediate N treatment results from a metabolic “sweet spot” where there is differential recycling of native proteins relative to Cel6A with less protein turnover at the highest N level and more tightly regulated protein synthesis at the lowest N treatment. Recombinant protein expression in plastid-engineered tobacco has previously been observed to alter native protein expression [20]. Transplastomic tobacco plants expressing either HPPD or GFP exhibited differential accumulation in 1–2% of endogenous proteins identified on a 2D-PAGE compared to WT tobacco; in particular, the transformed plants reduced expression of several Calvin cycle enzymes as well as glycine decarboxylase while up-regulating chaperones and peroxidases used in stress response [20]. More experimentation would be required to confirm similar disruptions in our TetC-*cel6*A plants and, then, subsequently optimize nutrient application for enzyme production.

Similar to our N experiments, we hypothesized that increased CO_2_ availability might increase foreign protein yield in our transgenic plants by increasing biomass and/or by altering protein allocation in the plant tissue [40]. Well-fertilized C3 plants grown in elevated CO_2_ often exhibit increased carbon fixation and reduced total Rubisco abundance [21, 22, 41]. Both of these metabolic changes should enhance foreign protein production. Indeed, the increase in CO_2_ concentration alleviated the stunted biomass phenotype in N-limited TetC-*cel6*A plants, and we observed an increase in Cel6A and “Other” native proteins concurrent with a significant decrease in Rubisco content. Previous studies have identified proteins involved in photosynthesis, hormone synthesis, and cell division as those “Other” native proteins most often differentially expressed in C3 plants grown in elevated CO_2_ (reviewed in [21]).

Thus, cultivating GE plants in high CO_2_ may be a viable method of alleviating deleterious phenotypes and enhancing foreign protein synthesis as a means of improving commercial viability of plastid-engineered plants for the production of certain target proteins. Greenhouse cultivation of our TetC-*cel6*A tobacco plants, assuming a 50-day growing period and supplemental lighting, yields raw production costs of approximately US$ 1.88 g^−1^ Cel6A [42]. This is roughly 30-fold higher than costs we calculated previously for field-grown TetC-*cel6*A [3] and would not be cost competitive with microbial fermenters at approximately $0.01 g^−1^ cellulase [43]. Greenhouse production would, however, be viable for production of higher value proteins such as medical peptides which are produced in mammalian expression systems at costs of up to US$300–3000 g^−1^ [44]. It would depend on the value of the protein whether the additional cost of elevating CO_2_ in the greenhouse to gain perhaps 30% higher foreign protein yields would be beneficial.

Effectively adapting chloroplast-engineering to produce novel proteins of interest, like high-value medical proteins, necessitates a stronger understanding of potential mechanisms of interaction between the recombinant protein and the host system. Here, we have identified some of these considerations as well as proposed methods of addressing common interferences with the native host system. For example, altered mutant phenotypes are not always caused by a depletion of protein resources but by the enzymatic activity of the recombinant protein interfering with host plant metabolism. Gray et al. (2009) documented that Cel6A activity in crude protein extracts was proportional to immunoblot density in their work with the T0 generation TetC-*cel6*A; therefore, we propose that Cel6A’s endoglucanase activity may be interfering with GA metabolism, leading to the minor mutant phenotypes observed in well-fertilized TetC-*cel6*A tobacco. The dwarf phenotype of our well-fertilized transgenic tobacco is likely due to a truncation of stem internode elongation rather than to reduced carbon metabolism because dry matter, biomass, and leaf area were all similar to WT when grown with no limiting environmental factors. This truncation, coupled with delayed germination, suggests that GA levels are diminished in our transgenic plants [33, 34]. Furthermore, increased water storage in our transgenic plants, reflected by elevated fresh weight-to-dry weight ratios in our N-limitation experiments (Additional file 6: Figure S4b), is also indicative of decreased GA [45].

Since a portion of GA synthesis occurs in the chloroplast, it is possible that Cel6A is enzymatically interfering with GA intermediates, although a mechanism for this interaction is not clear. However, because of their ability to hydrolyze ether bonds of carbohydrates, recombinant cellulases have been reported numerous times to interfere with native host plant metabolism, particularly in the carbohydrate-rich chloroplast [46, 47]. As noted earlier, one study suggested that their recombinant beta-glucosidase activated hormone conjugates that increased plant biomass [30], while another study attributed low biomass and leaf chlorosis to the carbohydrate-binding affinity of their recombinant cellulases [16]. Alternatively, reductions in GA synthesis enzymes associated with the reallocation of protein resources to Cel6A could reduce synthesis of bioactive GA.

It is also possible for specific elements of the transgenic cassette to generate mutant phenotypes independent of protein synthesis by interfering with endogenous RNA-binding proteins. For example, the use of an endogenous gene’s ribosome binding site (RBS) may alter native gene expression, as was suggested to explain the substantial decrease in Rubisco in plants expressing GFP and HPPD under control of the native *rbcL* RBS [9]. If ribosome binding at this RBS limits RbcL translation, then competition between the native and transgenic RBS could impede RbcL synthesis. Furthermore, the HPPD and GFP expression cassettes were both targeted for insertion adjacent to the native *rbcL,* which also may have impacted native expression of *rbcL.*

There are, however, other examples of high-accumulating transformants utilizing the *rbcL* leader sequence that do not exhibit reduced Rubisco [10]. This led us to consider whether downregulation of Rubisco in the GFP tobacco line may be due to codon use. If the codon use in the heterologous gene is very similar to RbcL, its expression may result in competition for charged tRNAs. Coding sequences utilizing many rare codons are often poorly expressed [48]; therefore, it is general practice to optimize codon use in heterologous genes to match the host system’s native codon bias, as was done for GFP [9]. However, because the plastid is derived from bacteria, prokaryotic genes are often used directly with no modification.

**Fig. 6:**
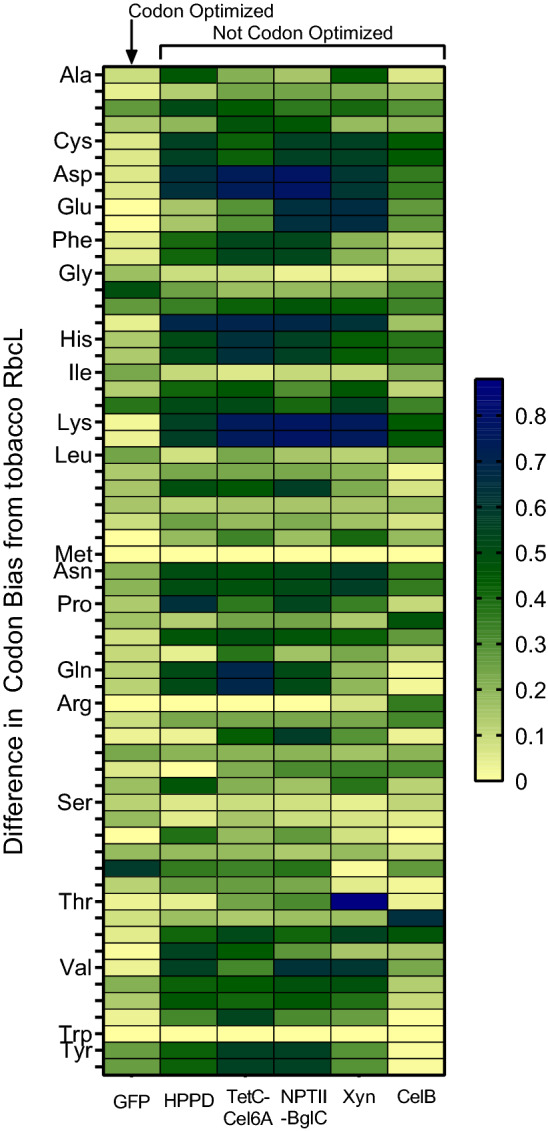
Summary of codon bias for several recombinant proteins compared to tobacco RbcL. Codon use (represented as proportions of each amino acid) of five different chloroplast-engineered recombinant genes was compared to that of *Nicotiana tabacum* RbcL to investigate the effect of codon optimization on Rubisco abundance in the transformed plants

To illustrate this point, we compared the codon use of several heterologous recombinant genes that were not codon optimized and the codon-optimized GFP to that of *Nicotiana tabacum’s* native *rbcL* (Fig. 6)*.* The unoptimized genes include, our own TetC-*cel6*A [12] and NPTII-*bglC* [13]*,* as well as another group’s recombinant xylanase and beta-glucosidase, CelB [10]. The GE plants containing these transgenes all achieved foreign protein yields of at least 30% of TSP, except NPTII-*bglC* which yielded approximately 15% of TSP and none of the engineered lines exhibited any deleterious mutant phenotypes.

Unsurprisingly, the codon use of the codon-optimized GFP is far more similar to RbcL than the genes that were not optimized. Rubisco content, quantified by immunoblots or estimated from SDS-PAGE images included in the original papers, was most substantially reduced in the GFP-expressing tobacco, moderately reduced in HPPD-expressing plants, and not reduced at all in tobacco expressing TetC-Cel6A, NPTII-BglC, Xylanase, or CelB. HPPD is a unique case because tobacco possesses an endogenous copy of HPPD, which may alter plant response to foreign HPPD expression. Of the subset of recombinant proteins that are truly foreign to tobacco, only the codon-optimized GFP suffered notable reductions in Rubisco. Thus, we propose that depressed expression of endogenous proteins can be specific to some component of the expression cassette, like the 5′UTR or RBS, or be caused by a more general diversion of resources.

## Conclusion

In this paper, we highlight some of the potential physiological challenges associated with producing a foreign protein to high levels in plant chloroplasts, but also explore interventions that could be implemented in the field or in customized plant growth chambers to ameliorate deleterious phenotypes. Consequently, these experiments with TetC-*cel6*A tobacco add to a growing body of work that demonstrates the feasibility of using plastid-engineered plants as a means of producing high-value proteins, potentially replacing the use of microbial and mammalian cell culture in some applications. Most notably, we demonstrated that cultivating over-burdened GE plants in elevated CO_2_ increases biomass, alleviates mutant phenotypes, and bolsters recombinant protein synthesis. We also presented a case for a recombinant protein enzymatically interfering with native metabolism. Finally, we offered some insights into other acute interactions between transgenic elements and the host plant system. Commercializing GE plants for high-value protein production relies on generating transgenic plants that produce and accumulate recombinant protein at high levels with reliably healthy plant growth. To accomplish this, we need to both better understand resultant alterations in plant physiology as well as to better understand plastid gene regulation. Both can lead to important insights about how to balance protein resource allocation with heterologous protein synthesis.

## Methods

### Cultivation of soil-grown tobacco

For the soil cultivation experiment, seeds were sown directly into 1-gallon pots containing soil and 10 g of 15–9-12 Osmocote fertilizer. Plants were watered as needed for the duration of the experiment with untreated tap water. The growth chamber was maintained on a 25 °C/20 °C day/night cycle with an 18-h day length. Chamber solar radiation was 300 micromoles m^−2^ day^−1^ and relative humidity was set to 70%. Plant trays were rotated every third day throughout the chamber to ensure that all plants received on average the same growth conditions.

Sixteen plants were grown for each genotype, WT and TetC-*cel6*A, and four individuals were randomly selected for harvest at each of four time points (2, 6, 9, and 12 weeks of age).

### Tissue harvesting

At harvest, the aboveground biomass was weighed, and tissue was collected for protein and dry weight analysis. Tissues samples averaging 4–8 cm^2^ leaf area for the CO_2_-ammonium nitrate trials and 2–6 cm^2^ for the age-dependence trial were collected from all green leaves of the plant. For 2-week-old seedlings of the age-dependence trial, the entire plant excluding the roots was harvested. All collected tissue was frozen in liquid nitrogen and stored at -80 °C until further analyses.

### Growth measurements

Stem length was measured from soil surface to apex with a tape measure. Measurements were taken six times throughout the duration of the experiment.

### Gibberellic acid treatment

Seeds were sterilized in 100% ethanol for 1 min and a 40% bleach, 0.5% SDS solution for 10 min before being rinsed four times in sterile water. Sterilized seeds were then placed evenly in two rows on a 15-cm diameter petri dish containing Murashige and Skoog media (3% sucrose) with either no GA_3_ (bioactive GA) or 1 µM GA_3_. The GA_3_ stock solution was prepared by dissolving powdered GA_3_ (Sigma-Aldrich) in 100% ethanol and filter-sterilized through a 0.2-µm filter.

Plates were then placed in the dark at 4 °C for 3 days before moving into a growth chamber with 16 h day length at 21 °C. Seeds were checked for root and cotyledon emergence every morning at the same time for 9 days. Plates were also photographed for shoot and root length measurements for a total of 24 days. These measurements were carried out on the same 16 representative seedlings in ImageJ [49] (version 1.52a).

### Cultivation of nitrogen-limited and CO_2_-enriched tobacco

WT and TetC-*cel6*A tobacco plants were grown in a Percival Scientific Inc. plant growth chamber (Iowa, USA) with 18-h days, 22 °C, 65% relative humidity, and illuminated with 280 micromoles m^−2^ day^−1^. For the elevated CO_2_ trial, CO_2_ was supplemented with Pure Clean CO_2_ (99.995% purity, Airgas, Pennsylvania, USA). Tobacco was seeded on soil and watered with fertilizer nutrient solution for 14 days before being transplanted into 4-inch-diameter round pots with autoclaved vermiculite. Six plants were grown per ammonium nitrate treatment for both genotypes. Plants were watered every 3 days with 50 mL of premade liquid nutrient solution (1, 4, or 8 mM NH_4_NO_3_, 4 mM CaSO_4_, 30 µM CaCl_2_, 250 µM KH_2_PO_4_, 750 µM MgSO_4_, 20 µM EDTA, 20 µM FeSO_4_, 2 µM ZnSO_4_, 0.5 µM CuSO_4_, 2 µM MnSO_4_, 0.5 µM Na_2_MoO_4_, and 42 µM H_3_BO_3_ [adapted from [41]). Six week old plants were harvested for data analysis after 4 weeks of the ammonium nitrate treatment. 

### Dry weight analyses

Weight measurements were made on two separate samples of all genotypes before and after drying the tissue. Approximately 200 mg of fresh tobacco leaf tissue was dried in pre-weighed and pre-dried envelopes.

### Carbon and nitrogen analysis

Total carbon and nitrogen were analyzed by the Cornell Nutrient Analysis Laboratory (Ithaca, NY) using a combustion analysis of dry tissue (*n* = 2).

### Leaf protein extraction

Frozen WT and TetC-*cel6*A leaf samples were manually ground on ice using a micropestle for 10 min (*n* = 3). A final volume of 500 µL (300 µL for 2-week-old seedlings) of protein extraction buffer (20 mM Tris–HCl, 1 mL Triton X-100, 0.1% SDS, 1.5 mM PMSF, and 0.001% β-mercaptoethanol) was added to the ground tissue. The samples were vortexed thoroughly before being centrifuged for 3 min at 20,000 × *g* at 4 °C. The supernatant was extracted, aliquoted, and stored at -80 °C until use.

### Total protein quantification

Total soluble protein from the tobacco tissue samples was quantified using a standard Bradford Assay. The BioRad Quick Start Bovine Serum Albumin Standard Set and 1X Dye Reagent (BioRad, Hercules, CA, USA) were used for all Bradford Assays. Tobacco samples were diluted with the extraction buffer described above to achieve a concentration within the linear range of the standard curve. Absorbance measurements were carried out on a BioTek Industries Synergy 4 plate reader (BioTek Industries, Winooski, VT, USA) at 595 nm.

### Immunoblotting

Protein samples and standards were mixed in a 1:1 ratio with a 2X Laemmli Sample Buffer (BioRad, Hercules, CA, USA), electrophoresed through 12% polyacrylamide gels and then transferred to PVDF membranes (BioRad, Hercules, CA, USA). Purified Rubisco protein (Agrisera, Vännäs, Sweden) was used for standard curve quantitation. Purified Cel6A and the anti-Cel6A primary antibody [50, 51] were supplied by the laboratory of the late professor David Wilson (Cornell University, Ithaca, NY, USA). The anti-Rubisco antibody was generously donated by Martin Parry (University of Lancaster, UK) [52, 53]. The secondary antibody used for all blots was a horseradish peroxidase-linked whole anti-rabbit IgG produced in donkey (GE Healthcare Life Sciences, Pittsburgh, PA, USA). All primary and secondary antibody treatments were diluted 1:20,000 in Antibody Signal Enhancer (Amresco, Solon, OH, USA).

Membranes were developed using a TMB stabilized substrate dye for horseradish peroxidase (Promega, Madison, WI, USA). Densitometry was performed using image analysis software (Integrated density in ImageJ [46]).

### Codon optimization

Transcript sequences were obtained for Beta-glucosidase CelB (NCBI Accession AF013169.2), endo-β-1,4-xylanase (NCBI Accession KJ466334) [10], HPPD [11] (NCBI Accession DQ459069.1), GFP [9] ( NCBI Accession EU870886.1), TetC-Cel6A [12], NPTII-BglC [13], and RbcL (NCBI Accession LT576836.1). Relative codon usage ratios were obtained using the Sequence Manipulation Suite: Codon Usage tool (http://www.bioinformatics.org/sms2/codon_usage.html). The absolute value of the difference of use proportion for each codon was compared between each test gene and *rbcL.*

### Statistical analysis

Statistics were analyzed using the JMP (Version 14.3). The Tukey–Kramer HSD test was used for pair-wise comparisons of the effects of age and genotype on various plant measurements in soil-grown tobacco as well as for comparisons of the effect of ammonium nitrate treatment for vermiculite-grown tobacco (alpha = 0.05). Tukey HSD was used for pair-wise comparisons of stem length by age, genotype, and GA treatment in the GA application trial (alpha = 0.05). Finally, two-tailed Student’s t-tests were used to compare differences in means of all single-factor comparisons, assuming unequal variances (alpha = 0.05). A full statistics summary can be found in Additional file 2: Table S1, although *p*-values of notable comparisons were included in figures, figure legends, and/or text.

## Supplementary Information

**Additional file 1: Figure S1.** Additional growth measurements of soil-grown transgenic tobacco. **a** Dry weight and **b** leaf area comparison between WT and TetC-*cel6*A tobacco by plant age. Bar heights and data points correspond to the mean and error bars reflect the standard error of the means. See "Methods" for a description of statistical analyses and Additional file 2: Table S1 contains a full detailed statistics report.

**Additional file 2: Table S1.** Statistical analysis of data for all growth trials.

**Additional file 3: Figure S2.** Protein accumulation normalized for whole plant biomass. **a** TSP and **b** Cel6A from seedling to senescent plant. **c** and **d** Depict total protein allocated to Rubisco, Cel6A, and “Other” endogenous proteins. Bar heights and data points correspond to the mean and error bars reflect the standard error of the means (*n* = 3). See "Methods" for a description of statistical analyses and Additional file 2: Table S1 contains a full detailed statistics report.

**Additional file 4: Figure S3.** Comparison of the effect of GA on average days until root emergence between genotypes. Bar heights correspond to the mean and error bars reflect the standard error of the means (*n* = 60). The *p*-values of notable comparisons are labeled with red asterisks, *p* < 0.001 (***). See "Methods" for a description of statistical analyses and Additional file 2: Table S1 contains a full detailed statistics report.

**Additional file 5: Figure S4.** Effect of enhanced CO_2_ on dry weight, water storage, and whole plant Cel6A accumulation. **a** Dry weight accumulation. **b** Water storage comparison between genotypes and CO_2_ treatment calculated as the ratio between fresh weight and dry weight. **c** Cel6A yield normalized to biomass. Bar heights correspond to the mean and error bars reflect the standard error of the means (*n* = 3). The *p*-values for notable comparisons between genotypes (red asterisks) and CO_2_ treatment (blue asterisks) are labeled as follows, *p* < 0.05 (*), *p* < 0.01 (**), and *p* < 0.001 (***). See "Methods" for a description of statistical analyses and Additional file 2: Table S1 contains a full detailed statistics report.

**Additional file 6: Table S2.** All data used for figures and statistical analyses presented in this manuscript.

## Data Availability

The datasets supporting the conclusions of this article are included within the article and Additional file 6: Table S2.

## Abbreviations

GE: Genetic engineering
TSP: Total soluble protein
UTR: Untranslated region
WT: Wildtype
GA: Gibberellic acid
N: Nitrogen
RBS: Ribosome binding site
ORF: Open reading frame
