# Supplementary figures and images for: Mitigation of deleterious phenotypes in chloroplast-engineered plants accumulating high levels of foreign proteins
Source: Biotechnol Biofuels. 2021 Feb 10;14:42. doi: 10.1186/s13068-021-01893-2 (PMC7877051; doi:10.1186/s13068-021-01893-2)

**a.**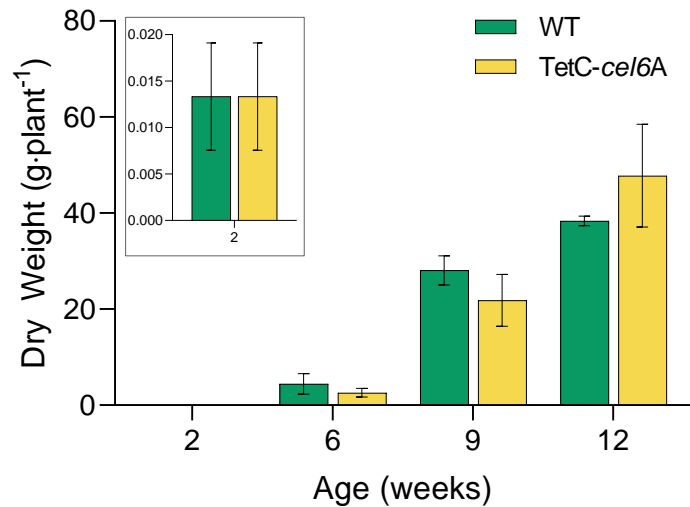**b.**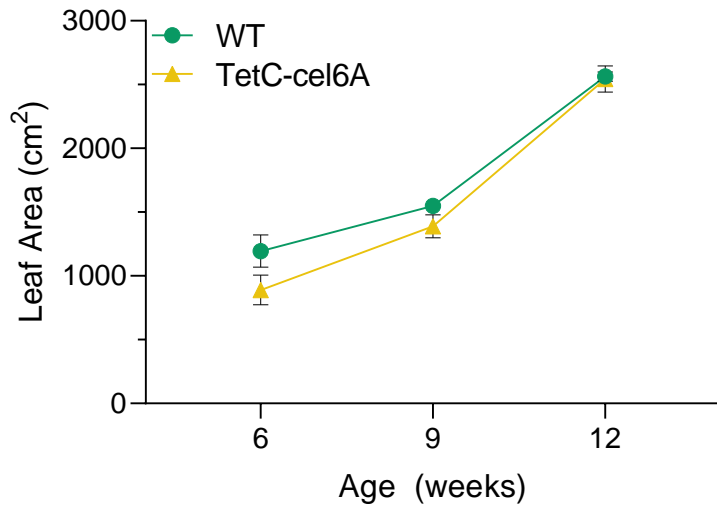

Supplement: Supplementary file 1 — Additional file 1: Figure S1. Additional growth measurements of soil-grown transgenic tobacco. a Dry weight and b leaf area comparison between WT and TetC-cel6A tobacco by plant age. Bar heights and data points correspond to the mean and error bars reflect the standard error of the means. See "Methods" for a description of statistical analyses and Additional file 2: Table S1 contains a full detailed statistics report. [file 13068_2021_1893_MOESM1_ESM.pdf]

**a.**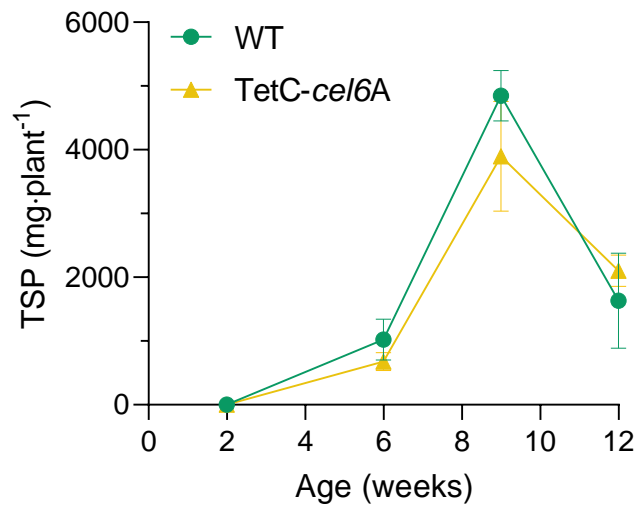**b.**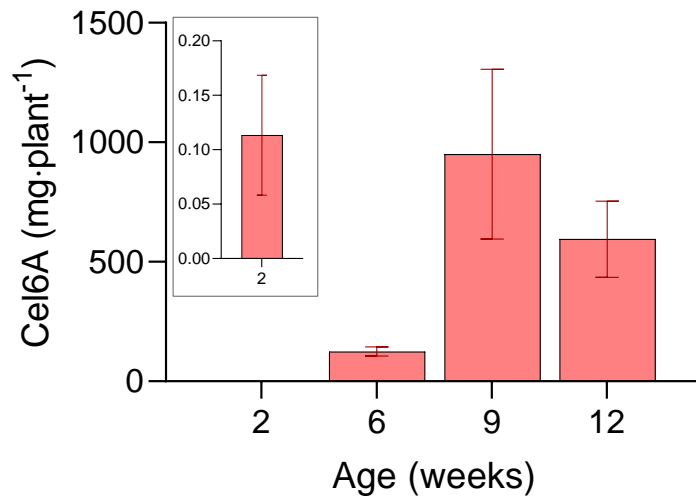**c.**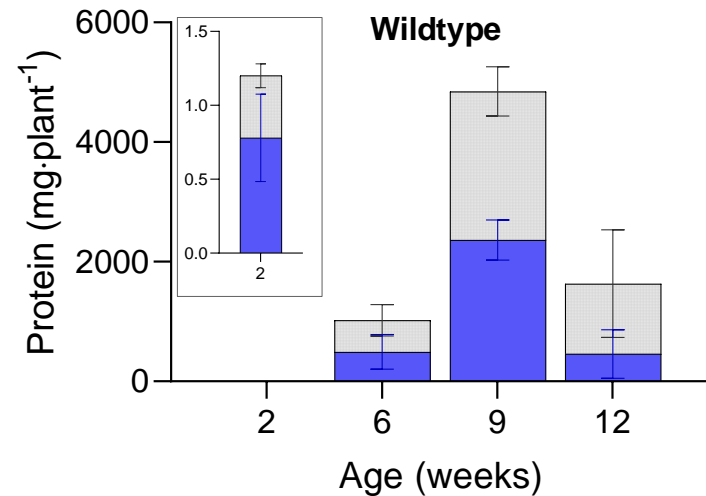**d.**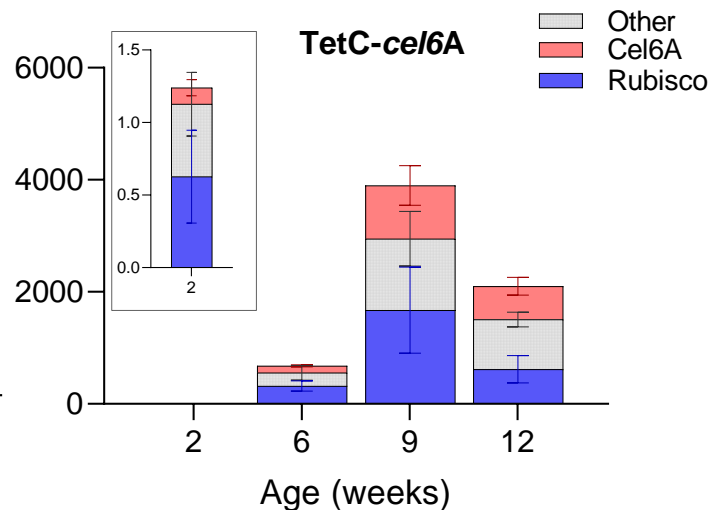

Supplement: Supplementary file 3 — Additional file 3: Figure S2. Protein accumulation normalized for whole plant biomass. a TSP and b Cel6A from seedling to senescent plant. c and d Depict total protein allocated to Rubisco, Cel6A, and “Other” endogenous proteins. Bar heights and data points correspond to the mean and error bars reflect the standard error of the means (n = 3). See "Methods" for a description of statistical analyses and Additional file 2: Table S1 contains a full detailed statistics report. [file 13068_2021_1893_MOESM3_ESM.pdf]

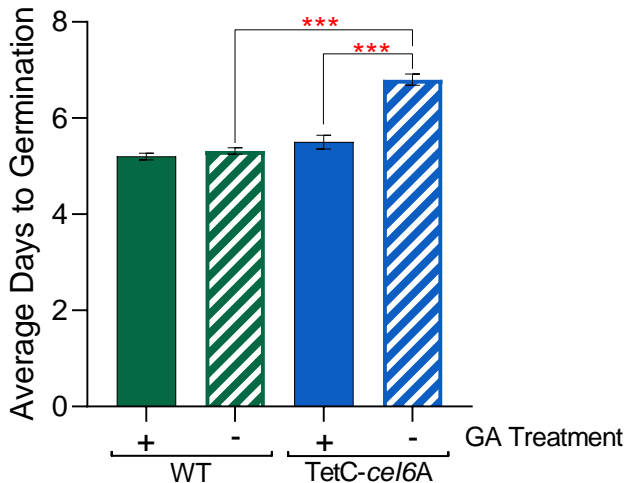

Supplement: Supplementary file 4 — Additional file 4: Figure S3. Comparison of the effect of GA on average days until root emergence between genotypes. Bar heights correspond to the mean and error bars reflect the standard error of the means (n = 60). The p-values of notable comparisons are labeled with red asterisks, p < 0.001 (***). See "Methods" for a description of statistical analyses and Additional file 2: Table S1 contains a full detailed statistics report. [file 13068_2021_1893_MOESM4_ESM.pdf]

**a.**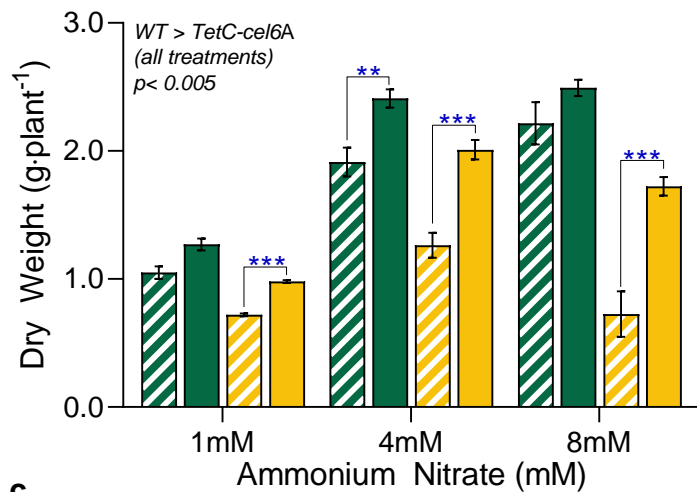**b.**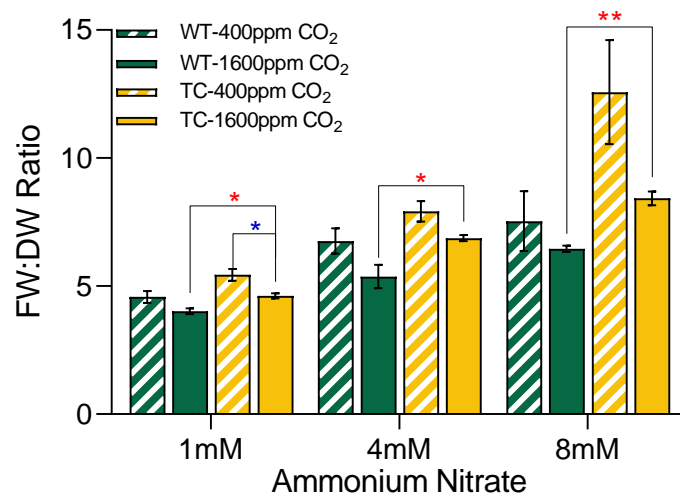**c.**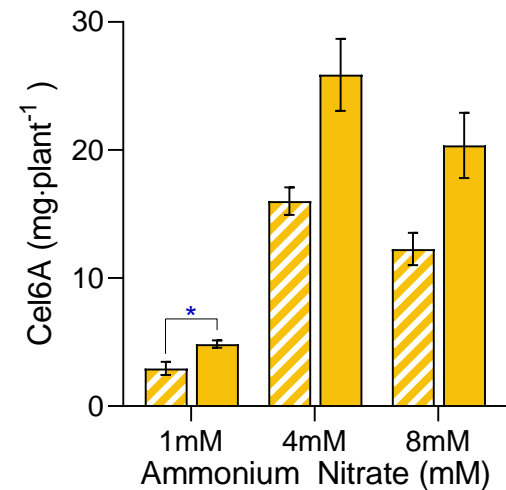

Supplement: Supplementary file 5 — Additional file 5: Figure S4. Effect of enhanced CO2 on dry weight, water storage, and whole plant Cel6A accumulation. a Dry weight accumulation. b Water storage comparison between genotypes and CO2 treatment calculated as the ratio between fresh weight and dry weight. c Cel6A yield normalized to biomass. Bar heights correspond to the mean and error bars reflect the standard error of the means (n = 3). The p-values for notable comparisons between genotypes (red asterisks) and CO2 treatment (blue asterisks) are labeled as follows, p < 0.05 (*), p < 0.01 (**), and p < 0.001 (***). See "Methods" for a description of statistical analyses and Additional file 2: Table S1 contains a full detailed statistics report. [file 13068_2021_1893_MOESM5_ESM.pdf]
