# Supplementary material for: Mitigation of deleterious phenotypes in chloroplast-engineered plants accumulating high levels of foreign proteins
Source: Biotechnol Biofuels. 2021 Feb 10;14:42. doi: 10.1186/s13068-021-01893-2 (PMC7877051; doi:10.1186/s13068-021-01893-2)
Supplement: Supplementary file 2 — Additional file 2: Table S1. Statistical analysis of data for all growth trials. [file 13068_2021_1893_MOESM2_ESM.pdf]

Age-dependence trial

Parameter	Treatment	Comparison		DF	t ratio	Lower CL dif	Upper CL dif	p-value
Fresh Weight	2 week old plants	WT	TetC-*cel6* A	15.527	2.072	0.00	0.16	0.0553
	6 week old plants	WT	TetC-*cel6* A	4.005	1.240	-30.19	78.94	0.2828
	9 week old plants	WT	TetC-*cel6* A	2.646	0.933	-74.58	130.13	0.428
	12 week old plants	WT	TetC-*cel6* A	2.460	0.975	-80.83	140.50	0.4155
	TetC-*cel6* A	2 week old plants	6 week old plants			11.64	97.53	0.0107
		6 week old plants	9 week old plants			154.08	264.97	<.0001
		9 week old plants	12 week old plants			-29.20	89.35	0.4874
	WT	2 week old plants	6 week old plants			45.56	112.20	<.0001
		6 week old plants	9 week old plants			173.71	252.13	<.0001
		9 week old plants	12 week old plants			-10.22	74.49	0.1737
Dry Weight	2 week old plants	WT	TetC-*cel6* A	4.000	0.000	-0.01	0.01	1
	6 week old plants	WT	TetC-*cel6* A	2.690	1.383	-2.69	6.37	0.2702
	9 week old plants	WT	TetC-*cel6* A	3.155	1.752	-4.79	17.27	0.1736
	12 week old plants	WT	TetC-*cel6* A	2.034	-1.521	-35.68	16.82	0.2657
TSP	2 week old plants	WT	TetC-*cel6* A	3.349	-0.092	-1.23	1.16	0.9318
(mg/plant)	6 week old plants	WT	TetC-*cel6* A	2.740	0.982	-831.90	1518.60	0.4048
	9 week old plants	WT	TetC-*cel6* A	2.809	1.002	-2181.40	4075.90	0.3948
	12 week old plants	WT	TetC-*cel6* A	2.434	-0.596	-3335.90	2398.90	0.6022
Rubisco	2 week old plants	WT	TetC-*cel6* A	3.865	3.409	2.40	25.14	0.0285
% TSP	6 week old plants	WT	TetC-*cel6* A	3.749	-0.292	-9.75	7.93	0.7854
	9 week old plants	WT	TetC-*cel6* A	2.896	1.585	-6.48	18.83	0.2143
	12 week old plants	WT	TetC-*cel6* A	3.737	-0.315	-24.57	19.69	0.7694
	TetC-*cel6* A	2 week old plants	6 week old plants			-13.22	17.84	0.9622
		6 week old plants	9 week old plants			-10.21	20.85	0.7011
		9 week old plants	12 week old plants			-1.66	29.40	0.0811
	WT	2 week old plants	6 week old plants			0.33	33.65	0.0458
		6 week old plants	9 week old plants			-14.90	18.42	0.9856
		9 week old plants	12 week old plants			5.83	39.15	0.0109
Rubisco	2 week old plants	WT	TetC-*cel6* A	3.974	0.610	-0.55	0.85	0.575
(mg/plant)	6 week old plants	WT	TetC-*cel6* A	2.401	0.984	-478.14	827.01	0.4135
	9 week old plants	WT	TetC-*cel6* A	2.742	1.428	-931.40	2310.10	0.2565
	12 week old plants	WT	TetC-*cel6* A	3.297	-0.586	-988.94	667.88	0.5953
	TetC-*cel6* A	2 week old plants	6 week old plants			-739.38	1376.79	0.7725
		6 week old plants	9 week old plants			296.31	2412.49	0.0146
		9 week old plants	12 week old plants			-4.22	2111.95	0.0509
	WT	2 week old plants	6 week old plants			-294.20	1280.18	0.2622
		6 week old plants	9 week old plants			1082.11	2656.49	0.0003
		9 week old plants	12 week old plants			1116.54	2690.92	0.0003
Cel6A	TetC-*cel6* A	2 week old plants	6 week old plants			2.87	18.24	0.0098
% TSP		6 week old plants	9 week old plants			-2.73	12.64	0.2425
		9 week old plants	12 week old plants			-4.17	11.19	0.4995
Cel6A	TetC-*cel6* A	2 week old plants	6 week old plants			-384.52	633.84	0.8597
mg/plant		6 week old plants	9 week old plants			316.73	1335.09	0.0037
		9 week old plants	12 week old plants			-153.21	865.15	0.1924
Cel6A	TetC-*cel6* A	2 week old plants	6 week old plants			0.02	0.06	0.0003
mg/mg		6 week old plants	9 week old plants			-0.01	0.03	0.494
		9 week old plants	12 week old plants			0.01	0.05	0.003
Stem length	27 days old	WT	TetC-*cel6* A			-9.08	10.03	0.9196
	40 days old	WT	TetC-*cel6* A			-3.06	16.06	0.1744
	54 days old	WT	TetC-*cel6* A			16.20	35.31	<.0001
	61 days old	WT	TetC-*cel6* A			25.70	44.81	<.0001
	68 days old	WT	TetC-*cel6* A			15.70	34.81	<.0001
	86 days old	WT	TetC-*cel6* A			5.33	27.08	0.0048

Germination and Shoot Elongation

Germination (seeds/day)

**GA Treatment**	**Comparison**		**DF**	**t ratio**	**Lower CL dif**	**Upper CL dif**	**p-value**
Minus	WT	TetC-*cel6* A	95.164	-10.816	-1.756	-1.211	<.0001
Plus	WT	TetC-*cel6* A	85.886	-1.875	-0.618	0.018	0.064
**Genotype**							
WT	Minus	Plus	117.963	-1.180	-0.312	0.079	0.240
TetC-*cel6* A	Minus	Plus	113.852	-6.986	-1.669	-0.931	<.0001

Gibberellic Acid Application

**GA**	**Age (day)**	**Comparison**		**Difference**	**Std Err Dif**	**Lower CL**	**Upper CL**	**p-value**
Minus	6	WT	TetC-*cel6* A	0.0003	0.026	-0.083	0.084	1
	10	WT	TetC-*cel6* A	0.010	0.025	-0.070	0.089	1
	12	WT	TetC-*cel6* A	0.034	0.025	-0.046	0.115	0.929
	17	WT	TetC-*cel6* A	0.091	0.025	0.010	0.173	0.016
	19	WT	TetC-*cel6* A	0.089	0.026	0.003	0.174	0.035
Plus	6	WT	TetC-*cel6* A	0.054	0.055	-0.119	0.226	0.978
	10	WT	TetC-*cel6* A	0.003	0.055	-0.169	0.176	1
	12	WT	TetC-*cel6* A	0.009	0.056	-0.164	0.183	1
	17	WT	TetC-*cel6* A	0.003	0.057	-0.175	0.180	1
Genotype	Age (day)							
WT	6	Minus	Plus	0.305	0.043	0.155	0.456	<.0001
	10	Minus	Plus	0.498	0.042	0.350	0.646	<.0001
	12	Minus	Plus	0.616	0.043	0.467	0.765	<.0001
	17	Minus	Plus	0.763	0.043	0.612	0.914	<.0001
TetC-*cel6* A	6	Minus	Plus	0.362	0.043	0.211	0.512	<.0001
	10	Minus	Plus	0.511	0.043	0.363	0.660	<.0001
	12	Minus	Plus	0.660	0.043	0.510	0.809	<.0001
	17	Minus	Plus	0.852	0.044	0.700	1.005	<.0001

CO2 and Ammonium Nitrate Trial

Parameter	Treatment 1	Treatment 2	Comparison		DF	t ratio	Lower CL dif	Upper CL dif	p-value
**Fresh Weight**	TetC-*cel6*A	Ambient CO2	8mM	1mM			2.89	9.23	0.0005
			4mM	1mM			1.68	8.02	0.0033
			8mM	4mM			-1.96	4.38	0.5945
		Elevated CO2	8mM	1mM			8.77	12.47	<.0001
			4mM	1mM			7.70	11.41	<.0001
			8mM	4mM			-0.78	2.92	0.3197
		1mM	Ambient CO2	Elevated CO2	9.89	2.78	0.13	1.20	0.0195
		4mM	Ambient CO2	Elevated CO2	9.24	5.61	3.21	7.52	0.0003
		8mM	Ambient CO2	Elevated CO2	7.35	3.67	1.89	8.56	0.0073
	WT	Ambient CO2	8mM	1mM			10.20	13.82	<.0001
			4mM	1mM			6.90	10.53	<.0001
			8mM	4mM			1.48	5.11	0.0008
		Elevated CO2	8mM	1mM			9.49	12.13	<.0001
			4mM	1mM			6.90	9.53	<.0001
			8mM	4mM			1.28	3.91	0.0003
	1mM	Ambient CO2	WT	TetC-cel6A	9.60	3.28	0.26	1.39	0.0087
		Elevated CO2	WT	TetC-cel6A	9.93	2.75	0.12	1.20	0.0207
	4mM	Ambient CO2	WT	TetC-cel6A	8.13	5.26	2.64	6.74	0.0007
		Elevated CO2	WT	TetC-cel6A	9.76	-0.90	-2.35	1.01	0.3918
	8mM	Ambient CO2	WT	TetC-cel6A	7.75	4.67	3.41	10.15	0.0017
		Elevated CO2	WT	TetC-cel6A	7.72	1.17	-0.83	2.53	0.2757
**Dry Weight**	TetC-*cel6*A	Ambient CO2	4mM	1mM			0.09	0.72	0.0111
			4mM	8mM			-0.05	0.58	0.1077
			8mM	1mM			-0.17	0.46	0.4766
		Elevated CO2	4mM	1mM			0.84	1.34	<.0001
			8mM	1mM			0.59	1.10	<.0001
			4mM	8mM			-0.01	0.50	0.0563
		1mM	Ambient CO2	Elevated CO2	9.36	5.36	0.15	0.37	0.0004
		4mM	Ambient CO2	Elevated CO2	9.77	7.36	0.66	1.23	<.0001
		8mM	Ambient CO2	Elevated CO2	9.01	7.33	0.66	1.26	<.0001
	WT	Ambient CO2	8mM	1mM			1.05	1.58	<.0001
			4mM	1mM			0.73	1.27	<.0001
			8mM	4mM			0.05	0.58	0.0211
		Elevated CO2	4mM	1mM			1.01	1.50	<.0001
			8mM	1mM			0.97	1.45	<.0001
			4mM	8mM			-0.20	0.29	0.8766
		1mM	Ambient CO2	Elevated CO2	9.97	4.36	0.13	0.39	0.0014
		4mM	Ambient CO2	Elevated CO2	9.21	4.47	0.25	0.77	0.0015
		8mM	Ambient CO2	Elevated CO2	7.80	1.39	-0.10	0.41	0.2017
	1mM	Ambient CO2	WT	TetC-cel6A	8.86	5.85	0.19	0.42	0.0003
		Elevated CO2	WT	TetC-cel6A	9.97	5.40	0.18	0.43	0.0003
	4mM	Ambient CO2	WT	TetC-cel6A	8.96	7.55	0.63	1.17	<.0001
		Elevated CO2	WT	TetC-cel6A	9.90	3.76	0.19	0.75	0.0038
	8mM	Ambient CO2	WT	TetC-cel6A	9.90	10.22	1.15	1.80	<.0001
		Elevated CO2	WT	TetC-cel6A	9.00	7.20	0.46	0.88	<.0001
**TSP**	TetC-*cel6*A	Ambient CO2	4mM	1mM			6.54	95.80	0.0291
**(mg/plant)**			4mM	8mM			0.53	89.78	0.0478
			8mM	1mM			51.70	140.96	0.0014
		Elevated CO2	4mM	1mM			31.22	120.95	0.0048
			8mM	1mM			58.58	148.32	0.0010
			4mM	8mM			-17.51	72.23	0.2269
		1mM	Ambient CO2	Elevated CO2	3.58	-1.25	-14.55	5.80	0.2864
		4mM	Ambient CO2	Elevated CO2	2.79	3.58	1.48	39.60	0.0419
		8mM	Ambient CO2	Elevated CO2	4.00	0.11	-64.88	70.37	0.9158
	WT	Ambient CO2	8mM	1mM			72.86	122.41	<.0001
			4mM	1mM			38.18	87.73	0.0006
			8mM	4mM			9.91	59.46	0.0121
		Elevated CO2	8mM	1mM			54.06	97.27	<.0001
			4mM	1mM			34.72	77.93	0.0005
			8mM	4mM			-2.26	40.94	0.0747
		1mM	Ambient CO2	Elevated CO2	3.06	8.22	10.19	22.83	0.0035
		4mM	Ambient CO2	Elevated CO2	3.60	0.97	-19.83	39.60	0.3947
		8mM	Ambient CO2	Elevated CO2	2.09	-0.69	-38.36	27.44	0.5610
	1mM	Ambient CO2	WT	TetC-cel6A	2.43	-2.74	-19.26	2.73	0.0899
		Elevated CO2	WT	TetC-cel6A	3.94	4.71	5.14	20.10	0.0096
	4mM	Ambient CO2	WT	TetC-cel6A	2.63	0.55	-18.48	25.51	0.6242
		Elevated CO2	WT	TetC-cel6A	3.36	-0.72	-36.71	22.43	0.5162
	8mM	Ambient CO2	WT	TetC-cel6A	2.78	-0.36	-70.56	56.64	0.7417
		Elevated CO2	WT	TetC-cel6A	2.02	-0.89	-87.89	57.57	0.4671

Parameter	Treatment 1	Treatment 2	Comparison		DF	t ratio	Lower CL dif	Upper CL dif	p-value
**Nitrogen**	TetC-*cel6*A	Ambient CO2	8mM	1mM	43.85	8.75	7.30	80.41	0.0309
**(mg/plant)**			4mM	1mM	33.19	8.75	-3.36	69.75	0.064
			8mM	4mM	10.66	8.75	-25.89	47.21	0.5212
		Elevated CO2	8mM	1mM	30.04	0.69	27.14	32.94	<.0001
			8mM	4mM	20.77	0.69	17.87	23.67	0.0002
			4mM	1mM	9.27	0.69	6.37	12.17	0.0019
		1mM	Ambient CO2	Elevated CO2	1.07	-4.32	-12.25	5.33	0.1329
		4mM	Ambient CO2	Elevated CO2	1.00	-2.56	-163.15	108.39	0.2372
		8mM	Ambient CO2	Elevated CO2	1.90	-52.70	-18.76	-15.79	0.0005
	WT	Ambient CO2	8mM	1mM	27.72	1.08	23.22	32.23	0.0003
			8mM	4mM	18.95	1.08	14.45	23.45	0.0008
			4mM	1mM	8.78	1.08	4.28	13.28	0.0079
		Elevated CO2	8mM	1mM	23.83	2.24	14.48	33.19	0.0036
			8mM	4mM	15.38	2.24	6.02	24.74	0.0129
			4mM	1mM	8.45	2.24	-0.90	17.81	0.0648
		1mM	Ambient CO2	Elevated CO2	1.13	-1.96	-8.24	5.45	0.2763
		4mM	Ambient CO2	Elevated CO2	1.12	-0.66	-27.57	24.14	0.6192
		8mM	Ambient CO2	Elevated CO2	1.98	-3.76	-11.39	0.82	0.065
	1mM	Ambient CO2	WT	TetC-cel6A	1.09	-2.95	-9.45	5.31	0.1913
		Elevated CO2	WT	TetC-cel6A	1.10	0.00	-8.28	8.27	0.9968
	4mM	Ambient CO2	WT	TetC-cel6A	1.01	-2.47	-160.68	107.70	0.2436
		Elevated CO2	WT	TetC-cel6A	1.02	-0.32	-31.76	30.11	0.8
	8mM	Ambient CO2	WT	TetC-cel6A	1.15	-18.63	-27.41	-8.99	0.023
		Elevated CO2	WT	TetC-cel6A	1.00	0.53	-134.84	146.69	0.6878
**Rubisco**	TetC-*cel6*A	Ambient CO2	1mM	4mM			7.93	18.56	0.0006
**% TSP**			8mM	4mM			2.70	13.33	0.0086
			1mM	8mM			-0.09	10.55	0.0532
		Elevated CO2	1mM	8mM	4.25	3.98	-7.97	16.48	0.5661
			4mM	8mM	3.38	3.98	-8.84	15.60	0.6895
			1mM	4mM	0.87	3.98	-11.35	13.10	0.9739
		1mM	Ambient CO2	Elevated CO2	2.15	-15.29	-37.89	-22.09	0.0031
		4mM	Ambient CO2	Elevated CO2	3.77	-5.25	-27.16	-8.07	0.0074
		8mM	Ambient CO2	Elevated CO2	2.04	-7.98	-44.34	-13.68	0.0144
	WT	Ambient CO2	8mM	1mM			-15.97	23.78	0.8238
			4mM	1mM			-16.96	22.79	0.8962
			8mM	4mM			-18.88	20.86	0.9872
		Elevated CO2	4mM	1mM			-8.55	29.39	0.2850
			4mM	8mM			-11.04	26.89	0.4543
			8mM	1mM			-16.47	21.46	0.9152
		1mM	Ambient CO2	Elevated CO2	2.40	-5.02	-34.13	-5.27	0.0253
		4mM	Ambient CO2	Elevated CO2	3.00	-1.98	-31.75	7.37	0.1416
		8mM	Ambient CO2	Elevated CO2	3.99	-2.58	-43.87	1.67	0.0617
	1mM	Ambient CO2	WT	TetC-cel6A	2.39	-9.68	-16.68	-7.47	0.0057
		Elevated CO2	WT	TetC-cel6A	2.99	-0.42	-15.17	11.61	0.7005
	4mM	Ambient CO2	WT	TetC-cel6A	2.56	0.70	-16.44	24.61	0.5417
		Elevated CO2	WT	TetC-cel6A	3.98	2.46	-1.26	20.29	0.0703
	8mM	Ambient CO2	WT	TetC-cel6A	2.02	-0.52	-27.02	21.15	0.6543
		Elevated CO2	WT	TetC-cel6A	3.30	0.71	-16.08	26.03	0.5224
**Rubisco**	TetC-*cel6*A	Ambient CO2	8mM	1mM			22.56	62.29	0.0015
**(mg/plant)**			8mM	4mM			7.32	47.05	0.0135
			4mM	1mM			-4.62	35.11	0.1229
		Elevated CO2	8mM	1mM			-2.52	37.16	0.0814
			4mM	1mM			-4.64	35.04	0.1235
			8mM	4mM			-17.72	21.96	0.9430
		1mM	Ambient CO2	Elevated CO2	3.35	-5.96	-14.74	-4.86	0.0069
		4mM	Ambient CO2	Elevated CO2	2.17	-2.61	-24.89	5.20	0.1109
		8mM	Ambient CO2	Elevated CO2	3.95	-3.35	-63.99	-5.82	0.0292
	WT	Ambient CO2	8mM	1mM			16.36	69.22	0.0061
			4mM	1mM			1.13	53.99	0.0426
			8mM	4mM			-11.20	41.66	0.2578
		Elevated CO2	4mM	1mM			0.60	41.20	0.0448
			8mM	1mM			-2.99	37.61	0.0881
			4mM	8mM			-16.71	23.89	0.8539
		1mM	Ambient CO2	Elevated CO2	2.10	-0.72	-7.29	5.11	0.5428
		4mM	Ambient CO2	Elevated CO2	3.22	-0.92	-33.63	18.13	0.4223
		8mM	Ambient CO2	Elevated CO2	3.94	-2.61	-54.96	1.82	0.0601
	1mM	Ambient CO2	WT	TetC-cel6A	2.12	-4.82	-12.61	-1.04	0.0362
		Elevated CO2	WT	TetC-cel6A	3.22	1.09	-3.40	7.17	0.3494

CO2 and Ammonium Nitrate Trial

Parameter	Treatment 1	Treatment 2	Comparison		DF	t ratio	Lower CL dif	Upper CL dif	p-value
	4mM	Ambient CO2	WT	TetC-cel6A	2.04	0.75	-25.44	36.42	36.4210
		Elevated CO2	WT	TetC-cel6A	3.92	1.35	-8.18	23.35	0.2508
	8mM	Ambient CO2	WT	TetC-cel6A	4.00	-0.59	-36.67	23.75	0.5844
		Elevated CO2	WT	TetC-cel6A	4.00	0.19	-25.02	28.76	0.8561
**Cel6A**	TetC-*cel6* A	Ambient CO2	4mM	1mM	9.96	0.99	6.92	13.00	0.0001
**% TSP**			4mM	8mM	9.91	0.99	6.87	12.95	0.0001
			8mM	1mM	0.05	0.99	-2.99	3.09	0.9986
		Elevated CO2	4mM	8mM	9.53	1.84	3.87	15.19	0.0050
			4mM	1mM	6.14	1.84	0.48	11.80	0.0363
			1mM	8mM	3.39	1.84	-2.27	9.05	0.2361
		1mM	Ambient CO2	Elevated CO2	2.98	5.17	3.61	15.28	0.0143
		4mM	Ambient CO2	Elevated CO2	3.04	3.20	0.07	11.18	0.0485
		8mM	Ambient CO2	Elevated CO2	3.29	15.94	4.87	7.15	0.0003
**Cel6A**	TetC-*cel6* A	Ambient CO2	4mM	1mM	13.07	1.41	8.73	17.41	0.0002
**mg/plant**			8mM	1mM	9.32	1.41	4.98	13.66	0.0014
			4mM	8mM	3.75	1.41	-0.59	8.09	0.0844
		Elevated CO2	4mM	1mM	21.03	3.11	11.48	30.58	0.0012
			8mM	1mM	15.51	3.11	5.96	25.06	0.0060
			4mM	8mM	5.52	3.11	-4.03	15.07	0.2563
		1mM	Ambient CO2	Elevated CO2	3.16	3.23	0.08	3.72	0.0449
		4mM	Ambient CO2	Elevated CO2	2.58	3.27	-0.68	20.40	0.0580
		8mM	Ambient CO2	Elevated CO2	2.91	2.84	-1.13	17.31	0.0680
