# Supplementary material for: Mitigation of deleterious phenotypes in chloroplast-engineered plants accumulating high levels of foreign proteins
Source: Biotechnol Biofuels. 2021 Feb 10;14:42. doi: 10.1186/s13068-021-01893-2 (PMC7877051; doi:10.1186/s13068-021-01893-2)
Supplement: Supplementary file 6 — Additional file 6: Table S2. All data used for figures and statistical analyses presented in this manuscript. [file 13068_2021_1893_MOESM6_ESM.pdf]

Age dependent biomass and protein data													
**Genotype**	**Age**	**Sample ID #**	**Fresh Weight**	**% Dry matter**	**Dry Weight**	**TSP**	**TSP**	**Rubisco**	**Rubisco**	**Cel6A**	**Cel6A**	**Cel6A**	**Other**
			**g/plant**		**g/plant**	**mg/mg**	**mg/plant**	**% TSP**	**mg/plant**	**% TSP**	**mg/mg**	**mg/plant**	**mg/plant**
				3	3	3	3	3	3	3	3	3	
WT	2	1	0.34										
		2	0.17										
		3	0.30										
		4	0.26	5.61	0.017	0.10	1.62	69.22	1.12				0.50
		5	0.24										
		6	0.08	6.75	0.011	0.090	0.97	64.51	0.63				0.34
		7	0.11										
		8	0.32	5.61	0.012	0.084	1.01	58.47	0.59				0.42
		9	0.25										
	6	10	60.65										
		11	59.57	4.31	2.57	0.17	448.2	43.65	195.6				252.6
		12	133.55	5.05	6.74	0.23	1555.5	50.16	780.2				775.3
		13	62.67	6.27	3.93	0.27	1065.8	47.43	505.5				560.3
	9	14	312.34	9.75	30.46	0.17	5237.4	45.55	2385.7				2851.7
		15	269.01	9.17	24.66	0.16	4055.3	49.73	2016.6				2038.7
		16	277.23										
		17	309.54	9.39	29.08	0.18	5243.1	51.25	2686.9				2556.2
	12	18	305.20	12.23	37.34	0.084	3119.0	28.99	904.1				2215.0
		19	328.80	11.70	38.48	0.020	757.7	14.61	110.7				647.0
		20	338.50	11.61	39.29	0.026	1024.0	35.47	363.2				660.9
TetC-*cel6* A	2	1	0.10	14.14	0.012	0.049	0.60	51.07	0.31	7.88	0.004	0.05	0.25
		2	0.08										
		3	0.08										
		4	0.20	8.82	0.016	0.083	1.36	45.50	0.62	11.10	0.009	0.15	0.59
		5	0.17										
		6	0.26	4.12	0.010	0.17	1.75	54.32	0.95	8.06	0.013	0.14	0.66
		7	0.25										
		8	0.20										
		9	0.10										
		10	0.06										
	6	11	37.00	4.42	1.64	0.25	402.4	52.81	212.5	25.48	0.063	102.53	87.34
		12	47.94										
		13	63.77	4.13	2.64	0.30	781.9	46.41	362.9	16.95	0.050	132.54	286.5
		14	70.23	4.89	3.43	0.25	855.1	44.74	382.6	16.28	0.041	139.24	333.3
	9	15	302.48	7.64	23.10	0.17	3824.8	35.72	1366.2	23.29	0.039	890.71	1567.9
		16	210.54	7.57	15.93	0.15	2447.7	45.34	1109.9	25.72	0.040	629.43	708.4
		17	279.75	9.46	26.45	0.20	5421.5	46.94	2545.1	24.57	0.050	1331.89	1544.5
	12	18	256.80	14.46	37.14	0.06	2109.7	36.24	764.4	28.18	0.016	594.54	750.7
		19	351.30	16.66	58.52	0.04	2526.6	30.05	759.3	29.83	0.013	753.68	1013.6
		20	274.90	17.36	47.73	0.03	1670.0	20.11	335.9	26.10	0.009	435.89	898.2

Age dependent stem height measurements		
**Genotype**	**Age**	**Stem Length**
	**(days)**	**(cm)**
WT	27	1.3
		1.5
		1.2
		1.1
	40	18.5
		18.8
		16.0
		13.5
	54	75.0
		73.0
		69.0
		67.0
	61	114.0
		103.0
		111.0
		113.0
	68	118.0
		105.0
		123.0
		131.0
	86	123.0
		126.0
		135.0
TetC-cel6A	27	0.7
		0.8
		0.8
		0.9
	40	8.0
		10.0
		13.3
		9.5
	54	38.0
		49.0
		53.0
		41.0
	61	63.0
		81.0
		87.0
		69.0
	68	85.0
		103.0
		104.0
		84.0
	86	115.0
		115.0
		108.0

Age dependent leaf area		
**Genotype**	**Age**	**(cm^2)**
WT	4 weeks	48.5
		40.0
		142.3
		37.4
		130.1
		109.2
		69.8
	6 weeks	976.6
		1193.1
		1413.7
	9 weeks	1578.3
		1562.8
		1631.4
		1417.7
	10 weeks	1976.2
		1666.8
		1702.3
	12 weeks	2606.7
		2491.2
		2591.6
TetC-*cel6* A	4 weeks	27.2
		36.5
		52.6
		49.3
		40.0
		75.7
		112.8
	6 weeks	724.9
		828.8
		1113.7
	9 weeks	1461.5
		1211.9
		1492.9
	10 weeks	2705.1
		2406.0
		1975.1
	12 weeks	2650.4
		2642.2
		2338.6

GA application experiment shoot length measurements							
**Genotype**	**GA Treatment**	**Sample #**	**Shoot Length (cm)**				
			**Day 11**	**Day 15**	**Day 17**	**Day 22**	**Day 24**
WT	0 uM	1	0.117	0.212		0.202	0.215
		2	0.089	0.205	0.246	0.505	0.544
		3		0.144	0.22	0.268	0.279
		4	0.13	0.189		0.276	
		5	0.126	0.226	0.283	0.437	0.426
		6	0.181	0.258	0.378	0.548	0.583
		7	0.136	0.197	0.275	0.56	0.553
		8	0.091		0.303	0.419	0.452
		9		0.249	0.34	0.462	0.469
		10	0.151	0.285	0.354	0.496	0.509
		11	0.123	0.24	0.245	0.405	0.373
		12	0.132	0.242	0.24	0.474	0.354
		13		0.268	0.262	0.456	0.456
		14	0.113	0.217	0.19		
		15	0.132	0.201	0.274	0.434	0.535
		16	0.122	0.204	0.248	0.426	0.449
	1 uM	1	0.42	0.606	0.784	1.144	
		2	0.483	0.575	0.819	1.077	
		3	0.412	0.713	0.823		
		4	0.438	0.809	0.944	1.33	
		5	0.384	0.729	0.865	1.283	
		6	0.514	0.896	1.037		
		7	0.45	0.753	0.904	1.243	
		8	0.474	0.673	0.888	1.023	
		9	0.413	0.79	0.917	1.226	
		10	0.441	0.874	1.028		
		11	0.454	0.78	0.962	1.284	
		12	0.501	0.929	1.085	1.309	
		13	0.398	0.597	0.821	1.099	
		14	0.396	0.637	0.916	1.224	
		15	0.423	0.654	0.702	1.045	
		16	0.282	0.515	0.63	0.943	
TetC-cel6A	0 uM	1		0.118	0.126	0.315	0.352
		2	0.115	0.225	0.272	0.436	0.344
		3	0.108	0.211	0.274	0.317	0.281
		4	0.185	0.249	0.272	0.371	0.381
		5	0.155	0.302	0.274	0.451	0.459
		6	0.09	0.201	0.179	0.46	0.479
		7	0.117	0.216	0.238	0.269	0.26
		8	0.159	0.208	0.219	0.199	0.221
		9		0.195	0.257	0.346	0.368
		10	0.087	0.206	0.236	0.227	
		11	0.152	0.253	0.208	0.345	
		12	0.173	0.247	0.32	0.343	0.398
		13	0.099	0.195	0.235	0.277	0.363
		14	0.106				
		15	0.079	0.161	0.106		
		16		0.215	0.275		
	1 uM	1	0.448	0.537	0.715	0.883	
		2	0.467	0.785	0.962	1.182	
		3	0.507	0.674	0.772	0.988	
		4	0.705	1.029	1.13	1.403	
		5	0.796	0.958			
		6	0.066	0.513	0.439	1.22	
		7	0.393	0.595	0.93	1.332	
		8	0.575	0.893	1.088	1.332	
		9	0.598	0.841	1.006	1.354	
		10	0.588	0.861	1.131	1.243	
		11	0.525	0.708	0.98	1.336	
		12	0.464	0.67	0.919		
		13	0.446	0.604	0.731	0.947	
		14	0.616	0.772	0.934	1.238	
		15	0.058	0.428	0.494	0.793	
		16	0.487	0.717	0.918	1.099	

WT germination rate data					
**[GA]**	**Sample #**	**Days to Germination**	**[GA]**	**Sample #**	**Days to Germination**
**0 uM**	1	5	**1 uM**	1	5
	2	6		2	5
	3	5		3	5
	4	5		4	5
	5	5		5	6
	6	5		6	5
	7	6		7	6
	8	5		8	5
	9	5		9	5
	10	6		10	6
	11	5		11	5
	12	5		12	6
	13			13	5
	14	5		14	6
	15	5		15	6
	16	6		16	6
	17	5		17	5
	18	5		18	5
	19	6		19	5
	20	7		20	5
	21	5		21	5
	22	6		22	5
	23	5		23	6
	24	5		24	5
	25	6		25	5
	26	5		26	4
	27	5		27	5
	28	5		28	5
	29	5		29	5
	30	6		30	7
	31	6		31	
	32	5		32	6
	33	5		33	5
	34	6		34	5
	35	5		35	5
	36			36	
	37	5		37	5
	38	5		38	5
	39	5		39	5
	40	5		40	5
	41	5		41	4
	42	6		42	5
	43	6		43	5
	44	6		44	5
	45	5		45	6
	46	5		46	6
	47	5		47	5
	48	5		48	5
	49	7		49	5
	50	5		50	5
	51	5		51	5
	52	5		52	5
	53	5		53	6
	54	5		54	4
	55	5		55	5
	56	5		56	5
	57	6		57	5
	58	5		58	5
	59	5		59	5
	60	5		60	5
	61	5		61	5
	62	6		62	6

TetC-*cel6* A germination rate data					
**[GA]**	**Sample #**	**Days to Germination**	**[GA]**	**Sample #**	**Days to Germination**
**0 uM**	1	6	**1 uM**	1	6
	2	7		2	5
	3	6		3	5
	4	6		4	4
	5			5	8
	6	6		6	10
	7	6		7	5
	8	6		8	7
	9	8		9	5
	10	6		10	
	11	8		11	4
	12	6		12	4
	13	6		13	5
	14	5		14	7
	15	6		15	7
	16	6		16	6
	17	8		17	4
	18	6		18	5
	19	6		19	6
	20	7		20	4
	21	6		21	6
	22	7		22	4
	23	6		23	5
	24	6		24	5
	25	6		25	7
	26	5		26	5
	27	6		27	4
	28	6		28	4
	29	9		29	5
	30	6		30	6
	31	6		31	4
	32	6		32	5
	33	7		33	5
	34	6		34	5
	35	6		35	5
	36	7		36	4
	37	6		37	5
	38	6		38	4
	39	6		39	4
	40	7		40	5
	41	7		41	4
	42	6		42	5
	43	6		43	4
	44	7		44	4
	45	6		45	4
	46	6		46	5
	47			47	6
	48	6		48	4
	49	7		49	5
	50	6		50	4
	51	5		51	5
	52	7		52	8
	53	6		53	6
	54	6		54	4
	55	8		55	4
	56	7		56	5
	57	6		57	5
	58	5		58	5
	59	7		59	5
	60	6		60	4
	61	5		61	8
	62	6		62	5

Data report for CO_2 and ammonium nitrate growth chamber trial																		
Genotype	CO_2	Ammonium Nitrate	Fresh Weight	Fresh Weight	% Dry matter	Dry Weight	Carbon	Carbon	Carbon	Nitrogen	Nitrogen	Nitrogen	TSP	Rubisco	Rubisco	Cel6A	Cel6A	Other
			g/plant	g/plant/mM		g/plant	%	g/plant	g/plant/mM	%	mg/plant	mg/plant/mM	mg/plant	% TSP	mg/plant	% TSP	mg/plant	mg/plant
WT	Ambient	1mM	4.84	4.84	20.05	1.06		0.45	0.45		10.27	10.27						
			4.12	4.12		0.90	42.53	0.38	0.38	1.04	8.74	8.74	23.46	37.48	8.79			14.67
			5.18	5.18	23.91	1.13		0.48	0.48		10.99	10.99						
			4.29	4.29		0.94	42.76	0.40	0.40	0.90	9.10	9.10	21.02	37.89	7.97			13.06
			4.37	4.37	21.88	0.96		0.41	0.41		9.27	9.27						
			3.88	3.88		0.85		0.36	0.36		8.23	8.23	20.33	41.24	8.39			11.95
		4mM	11.29	2.82	13.28	1.69		0.74	0.18		31.27	7.82						
			14.11	3.53		2.12	43.87	0.92	0.23	1.91	39.08	9.77	76.01	36.89	28.04			47.97
			13.59	3.40	17.15	2.04		0.89	0.22		37.64	9.41						
			12.41	3.10		1.86	43.49	0.81	0.20	1.78	34.37	8.59	95.91	52.68	50.52			45.39
			14.17	3.54		2.13		0.93	0.23		39.25	9.81						
			13.40	3.35	14.45	2.01		0.88	0.22		37.12	9.28	81.76	35.78	29.27			52.49
		8mM	16.85	2.11	14.57	2.34		1.06	0.13		87.63	10.95						
			15.28	1.91		2.12		0.96	0.12		79.46	9.93	108.30	33.35	36.12			72.19
			13.59	1.70	16.84	1.89		0.85	0.11		70.67	8.83						
			18.16	2.27		2.52	44.87	1.14	0.14	3.65	94.44	11.80	134.52	42.15	56.70			77.82
			17.39	2.17	10.20	2.42		1.09	0.14		90.43	11.30						
			17.47	2.18		2.43	45.46	1.10	0.14	3.84	90.85	11.36	114.91	52.82	60.70			54.21
	Elevated	1mM	5.33	5.33		1.33		0.56	0.56		11.01	11.01						
			5.29	5.29	26.32	1.32		0.56	0.56		10.93	10.93	39.51	25.69	10.15			29.36
			4.52	4.52		1.13		0.48	0.48		9.34	9.34						
			5.28	5.28	24.43	1.31	42.22	0.56	0.56	0.81	10.91	10.91	34.60	19.07	6.60			28.00
			4.51	4.51		1.12		0.47	0.47		9.31	9.31						
			4.73	4.73	24.04	1.18	42.28	0.50	0.50	0.85	9.77	9.77	40.24	12.76	5.13			35.11
		4mM	13.99	3.50		2.64		1.14	0.29		44.28	11.07						
			12.07	3.02	16.33	2.28	43.21	0.98	0.25	1.93	38.21	9.55	107.93	29.09	31.40			76.53
			11.79	2.95		2.23		0.96	0.24		37.32	9.33						
			13.31	3.33	18.38	2.52		1.08	0.27		42.13	10.53	96.26	34.72	33.42			62.84
			14.97	3.74		2.83		1.22	0.30		47.39	11.85						
			12.84	3.21	21.96	2.43	43.03	1.05	0.26	1.42	40.64	10.16	79.14	24.97	19.76			59.38
		8mM	14.33	1.79		2.22		0.97	0.12		71.36	8.92						
			15.37	1.92	15.78	2.38	43.04	1.04	0.13	3.11	76.54	9.57	114.74	29.68	34.06			80.69
			16.26	2.03		2.52		1.10	0.14		80.97	10.12						
			16.75	2.09	14.91	2.60	43.90	1.13	0.14	3.32	83.41	10.43	111.47	25.28	28.18			83.29
			15.66	1.96		2.43		1.06	0.13		77.98	9.75						
			16.15	2.02	15.82	2.50		1.09	0.14		80.42	10.05	115.14	10.05	11.57			103.57
TetC-cel6A	Ambient	1mM	3.99	3.99	19.75	0.74		0.31	0.31		8.68	8.68						
			3.17	3.17		0.59	41.86	0.25	0.25	1.19	6.90	6.90	29.08	51.64	15.02	8.44	2.45	11.61
			3.88	3.88	18.52	0.72		0.30	0.30		8.44	8.44						
			3.81	3.81		0.70	42.15	0.30	0.30	1.16	8.29	8.29	35.18	50.35	17.71	11.29	3.97	13.49
			3.78	3.78	17.09	0.70		0.29	0.29		8.22	8.22						
			3.08	3.08		0.57		0.24	0.24		6.70	6.70	25.35	50.83	12.89	9.55	2.42	10.04
		4mM	10.21	2.55	11.58	1.30		0.58	0.14		58.29	14.57						
			7.48	1.87		0.95	43.61	0.42	0.11	3.42	42.70	10.68	85.06	34.46	29.31	20.46	17.41	38.34
			11.07	2.77	12.68	1.41		0.63	0.16		63.20	15.80						
			7.49	1.87		0.95		0.42	0.11		42.76	10.69	81.23	37.12	30.15	20.63	16.75	34.32
			8.54	2.14	13.83	1.08		0.48	0.12		48.76	12.19						
			6.03	1.51		0.77	45.61	0.34	0.09	5.57	34.43	8.61	76.85	41.51	31.90	18.08	13.89	31.06
		8mM	9.19	1.15	8.36	0.77		0.35	0.04		42.93	5.37						
			13.86	1.73		1.16	45.92	0.53	0.07	5.54	64.75	8.09	161.00	45.45	73.17	9.18	14.78	73.04
			4.82	0.60	6.11	0.40		0.19	0.02		22.52	2.81						
			9.90	1.24		0.83		0.38	0.05		46.25	5.78	110.41	45.23	49.94	10.00	11.04	49.43
			12.00	1.50	10.64	1.01		0.46	0.06		56.06	7.01						
			8.30	1.04		0.70	45.78	0.32	0.04	5.59	38.77	4.85	107.20	46.45	49.79	10.25	10.99	46.42
	Elevated	1mM	4.63	4.63	22.56	1.00		0.42	0.42		8.34	8.34						
			3.94	3.94		0.85	41.80	0.36	0.36	0.91	7.10	7.10	28.02	24.57	6.89	19.25	5.39	15.74
			4.43	4.43	21.07	0.96		0.40	0.40		7.98	7.98						
			3.56	3.56		0.77		0.32	0.32		6.41	6.41	21.53	18.01	3.88	22.00	4.74	12.92
			4.53	4.53	21.39	0.98		0.41	0.41		8.16	8.16						
			4.60	4.60		1.00	41.59	0.42	0.42	0.75	8.28	8.28	26.94	20.28	5.46	16.37	4.41	17.07
		4mM	14.60	3.65	14.99	2.13		0.92	0.23		37.45	9.36						
			16.10	4.03		2.35	43.31	1.01	0.25	1.78	41.30	10.33	110.49	25.33	27.98	28.44	31.42	51.09
			12.82	3.21	14.53	1.87		0.80	0.20		32.89	8.22						
			12.13	3.03		1.77		0.76	0.19		31.12	7.78	101.86	16.80	17.11	23.50	23.93	60.81
			13.89	3.47	14.14	2.03		0.87	0.22		35.63	8.91						
			13.47	3.37		1.97	42.70	0.85	0.21	1.73	34.56	8.64	92.40	18.11	16.73	24.11	22.28	53.39
		8mM	13.26	1.66	11.40	1.58		0.69	0.09		60.50	7.56						
			17.76	2.22		2.11	44.22	0.93	0.12	3.85	81.03	10.13	96.67	9.50	9.18	15.91	15.38	72.11
			15.09	1.89	12.67	1.80		0.79	0.10		68.85	8.61						
			14.18	1.77		1.69		0.74	0.09		64.70	8.09	135.73	19.72	26.77	16.10	21.85	87.11
			15.07	1.88	11.60	1.79		0.79	0.10		68.76	8.59						
			14.06	1.76		1.67	43.50	0.73	0.09	3.81	64.15	8.02	154.43	20.87	32.23	15.44	23.85	98.34

Codon Usage								
**Amino Acid**	**Codon**	**RbcL**	**TetC-Cel6A**	**NPTII-BglC**	**HPPD**	**GFP**	**Xylanase**	**CelB**
Ala	GCG	0.09	0.3	0.24	0.54	0	0.53	0.03
Ala	GCA	0.26	0.02	0.02	0.13	0.22	0.05	0.42
Ala	GCT	0.51	0.07	0.15	0	0.78	0.11	0.22
Ala	GCC	0.14	0.61	0.59	0.33	0	0.32	0.33
Cys	TGT	0.56	0.14	0	0	0.5	0	1
Cys	TGC	0.44	0.86	1	1	0.5	1	0
Asp	GAT	0.88	0.14	0.1	0.24	0.94	0.27	0.53
Asp	GAC	0.12	0.86	0.9	0.76	0.06	0.73	0.47
Glu	GAG	0.25	0.54	0.9	0.4	0.25	0.91	0.52
Glu	GAA	0.75	0.46	0.1	0.6	0.75	0.09	0.48
Phe	TTT	0.53	0	0	0.13	0.58	0.33	0.63
Phe	TTC	0.47	1	1	0.88	0.42	0.67	0.38
Gly	GGG	0.17	0.08	0.14	0.08	0	0.2	0.06
Gly	GGA	0.28	0.11	0.1	0.03	0.77	0.07	0.57
Gly	GGT	0.5	0.08	0.04	0.16	0.23	0.07	0.17
Gly	GGC	0.04	0.74	0.72	0.73	0	0.67	0.2
His	CAT	0.64	0	0.08	0.13	0.5	0.21	0.27
His	CAC	0.36	1	0.92	0.88	0.5	0.79	0.73
Ile	ATA	0.1	0.04	0	0	0.33	0	0.32
Ile	ATT	0.45	0	0.15	0.04	0.58	0	0.56
Ile	ATC	0.45	0.96	0.85	0.96	0.08	1	0.12
Lys	AAG	0.13	0.88	0.9	0.69	0.15	0.88	0.57
Lys	AAA	0.88	0.13	0.1	0.31	0.85	0.13	0.43
Leu	TTG	0.23	0	0.08	0.15	0.47	0.11	0.03
Leu	TTA	0.23	0	0	0	0.37	0.03	0.21
Leu	CTG	0.15	0.6	0.72	0.64	0	0.37	0.08
Leu	CTA	0.15	0	0	0.03	0	0	0.33
Leu	CTT	0.25	0.07	0.03	0	0.16	0.09	0.18
Leu	CTC	0	0.33	0.17	0.18	0	0.4	0.18
Met	ATG	1	1	1	1	1	1	1
Asn	AAT	0.57	0.09	0.06	0.07	0.77	0	0.22
Asn	AAC	0.43	0.91	0.94	0.93	0.23	1	0.78
Pro	CCG	0.14	0.5	0.68	0.78	0	0.48	0.04
Pro	CCA	0.24	0	0	0.11	0.4	0.1	0.71
Pro	CCT	0.52	0.03	0.06	0.06	0.6	0.1	0.25
Pro	CCC	0.1	0.47	0.26	0.06	0	0.33	0
Gln	CAG	0.31	1	0.82	0.82	0.43	0.5	0.33
Gln	CAA	0.69	0	0.18	0.18	0.57	0.5	0.67
Arg	AGG	0	0	0	0	0	0.07	0.35
Arg	AGA	0.23	0	0	0	0.14	0	0.55
Arg	CGG	0.03	0.46	0.61	0	0	0.32	0
Arg	CGA	0.2	0	0	0	0.43	0.04	0
Arg	CGT	0.37	0.15	0.06	0.37	0.43	0.04	0.05
Arg	CGC	0.17	0.38	0.33	0.63	0	0.54	0.05
Ser	AGT	0.12	0.03	0.04	0.06	0	0.08	0.23
Ser	AGC	0.18	0.33	0.27	0.13	0	0.25	0.23
Ser	TCG	0	0.18	0.27	0.38	0	0.08	0
Ser	TCA	0.18	0	0.04	0	0	0	0.27
Ser	TCT	0.41	0.08	0.04	0.06	1	0.42	0.14
Ser	TCC	0.12	0.38	0.35	0.38	0	0.17	0.14
Thr	ACG	0.03	0.27	0.34	0.07	0	0.91	0
Thr	ACA	0.17	0.03	0	0	0.25	0	0.82
Thr	ACT	0.55	0.03	0.14	0.14	0.5	0	0.09
Thr	ACC	0.24	0.68	0.52	0.79	0.25	0.09	0.09
Val	GTG	0.03	0.35	0.66	0.59	0	0.64	0.26
Val	GTA	0.48	0.04	0	0.06	0.69	0	0.35
Val	GTT	0.45	0.04	0	0	0.31	0.07	0.35
Val	GTC	0.03	0.57	0.34	0.35	0	0.29	0.03
Trp	TGG	1	1	1	1	1	1	1
Tyr	TAT	0.56	0	0	0.14	0.82	0.27	0.55
Tyr	TAC	0.44	1	1	0.86	0.18	0.73	0.45
End	TGA	0	1	0	0	0	1	0
End	TAG	0	0	1	0	0	0	1
End	TAA	0	0	0	1	1	0	0

Calculated from http://www.bioinformatics.org/sms2/codon_usage.html
